# Lnc‐HZ05 Suppresses Trophoblast Cell Migrasome Formation by Disrupting TGFβ2 Pathway in Unexplained Recurrent Miscarriage

**DOI:** 10.1002/advs.202417558

**Published:** 2025-10-30

**Authors:** Weina Chen, Ying Zhang, Zimeng Zhao, Yang Yang, Geng Guo, Yi Sun, Tao Zhang, Zhihong Zhang, Liqin Zeng, Wenxin Huang, Qingjun Shen, Ruining Han, Zhaohui He, Depeng Zhao, Huidong Zhang

**Affiliations:** ^1^ Research Center for Environment and Female Reproductive Health The Eighth Affiliated Hospital Sun Yat‐sen University Shenzhen 518033 China; ^2^ Department of Endocrinology Metabolic and Chronic Disease Science Innovation Center The Second Affiliated Hospital of Army Medical University Chongqing 400037 China; ^3^ Shenzhen Maternity and Child Healthcare Hospital Southern Medical University Shenzhen Guangdong 518028 China; ^4^ Reproductive Medical Center The First Affiliated Hospital of Sun Yat‐sen University Guangzhou Guangdong 510080 China; ^5^ Department of Obstetrics/Gynecology Joint Laboratory of Reproductive Medicine, West China Second University Hospital Sichuan University Chengdu 610041 China; ^6^ Department of Emergency Cerebrovascular disease center First Hospital of Shanxi Medical University Taiyuan 030001 China; ^7^ Department of Obstetrics and Gynaecology, Faculty of Medicine The Chinese University of Hong Kong Hong Kong 999077 China; ^8^ MOE Key Laboratory of Coal Environmental Pathogenicity and Prevention Shanxi Medical University Taiyuan 030001 China; ^9^ Department of Obstetrics The Eighth Affiliated Hospital Sun Yat‐Sen University Shenzhen Guangdong Province 518033 China; ^10^ Department of Urology, The Eighth Affiliated Hospital Sun Yat‐Sen University Shenzhen 518033 China

**Keywords:** female trophoblast cells, LncRNA or lnc‐HZ05, migration/invasion and migrasome formation, miscarriage, TGFβ2/TGFβR2/Smad3/FOXP3

## Abstract

Unexplained recurrent miscarriage (RM) is a clinical challenge due to its unclear pathogenesis. TGFβ2 plays essential roles in reproductive events. Migrasomes are newly identified organelles. Herein, whether TGFβ2 might regulate trophoblast cell migrasome formation (MF) and miscarriage, and the epigenetic regulation mechanisms, is completely unknown. In this study, we find that TGFβ2‐mediated MF is suppressed and is negatively associated with RM based on a case‐control study, further confirmed by a mouse model with miscarriage. In cellular mechanism, TGFβ2 promotes trophoblast cell MF, specifically suppressed by lnc‐HZ05. In details, lnc‐HZ05 (1) suppresses FOXP3‐mediated TGFβ2 transcription, (2) promotes autophagy degradation of TGFβ2, and (3) impairs TGFβ2/TGFβR2 interactions by binding to both proteins with 1‐83 nt of lnc‐HZ05, three of which suppress TGFβ2 pathway. Meanwhile, DNMT1 suppresses FOXP3‐mediated lnc‐HZ05 transcription, forming a FOXP3/lnc‐HZ05 negative regulatory loop. The cellular mechanisms are consistent with those in RM villous tissues. Moreover, higher levels of TGFβ2 protein and lnc‐HZ05 in serum well predict miscarriage risk. Supplement with murine Tgfβ2 protein recovers MF and alleviates mouse miscarriage. Collectively, this study discovers novel biological mechanisms of lnc‐HZ05 and TGFβ2 pathway in the pathogenesis of RM and provides potential targets for prediction and therapy of RM.

## Graphical abstract

TGFβ2 signaling‐mediated migration/invasion and migrasome formation are suppressed in recurrent miscarriage (RM) versus healthy control villous tissues and are negatively associated with unexplained RM. In mechanism, TGFβ2 promotes trophoblast cell migration/invasion and migrasome formation, all of which are suppressed by lnc‐HZ05. In details, lnc‐HZ05 suppresses FOXP3‐mediated TGFβ2 mRNA transcription, promotes autophagy degradation of TGFβ2 protein, and impairs TGFβ2/TGFβR2 protein interactions.

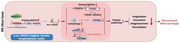

## 1 Introduction

*Recurrent miscarriage is related with dysfunctions of trophoblast cells*. ≈15–25% of pregnant women might experience miscarriage; and 1–5% might suffer from recurrent miscarriage (RM, two or more consecutive spontaneous miscarriage).^[^ ^1^ ^]^ Miscarriage or RM greatly limits global human reproduction. Moreover, ≈41% of RM patients might experience anxiety, 9% suffer from major depression, and 1.4% involve in death.^[^ ^2^ ^]^ In general, genetic, structural, infective, endocrine, immune, or thrombophilic disorders could cause or induce miscarriage.^[^ ^3^ ^]^ However, there are still 50–60% of RM with unexplained reasons,^[^ ^4^, ^5^ ^]^ which greatly impedes its effective treatment. During pregnancy, extravillous trophoblast cells play important roles in embryo implantation and placenta development.^[^ ^6^ ^]^ Dysfunctions of migration/invasion of human trophoblast cells may lead to adverse pregnancy outcomes, such as miscarriage.^[^ ^7^, ^8^, ^9^ ^]^ However, the underlying mechanisms how trophoblast cell migration/invasion is suppressed are still largely elusive.

*Trophoblast cell migrasome formation might be related with miscarriage*. Migrasomes are newly identified organelles with vesicle‐like morphology produced by migrating cells.^[^ ^10^ ^]^ Migrasome formation strictly depends on cell migration.^[^ ^11^ ^]^ It has been reported that migrasomes govern critical cellular processes such as mitochondrial quality control,^[^ ^12^ ^]^ cell‐cell communications,^[^ ^13^ ^]^ and lateral transfer of mRNAs and proteins,^[^ ^14^ ^]^ and also associated with various diseases, such as neurological disorders,^[^ ^15^ ^]^ cardiovascular diseases,^[^ ^16^ ^]^ kidney diseases,^[^ ^17^ ^]^ and ophthalmic conditions.^[^ ^18^ ^]^ NDST1 (bifunctional heparan sulfate N‐deacetylase/N‐sulfotransferase 1) and TSPAN4 (tetraspanin 4) take crucial roles in migrasome formation and could be considered as key indicators for migrasome formation.^[^ ^12^, ^19^, ^20^ ^]^ However, the mechanisms for migrasome formation are still largely elusive. Moreover, whether and how trophoblast cell migrasome formation might be associated with unexplained miscarriage is also largely unexplored.

*TGFβ2 signaling might be associated with trophoblast cell migrasome formation and miscarriage*. It has been reported that TGFβ (transforming growth factor beta) regulates numerous cellular processes, such as cell proliferation, migration, autophagy, apoptosis, and senescence.^[^ ^21^ ^]^ TGFβ pathway could be activated by TGF‐βs (TGFβ1, TGFβ2, and TGFβ3), which are secreted by cells and have similar but not identical functions.^[^ ^22^, ^23^ ^]^ TGF‐β binds to protein kinase receptors TGFβR1 or TGFβR2, which subsequently induces phosphorylation and activation of its downstream Smad2 or Smad3.^[^ ^24^ ^]^ Then, Smads re‐enter nucleus and activate or repress the transcription of hundreds of genes to regulate various cell phenotypes.^[^ ^24^ ^]^ Increasing evidence has demonstrated that TGFβ signaling is involved in many fundamental reproductive events, such as invasion,^[^ ^25^ ^]^ decidualization,^[^ ^26^ ^]^ maternal‐embryo communication,^[^ ^27^ ^]^ and embryonic development.^[^ ^28^ ^]^ Exogenous TGFβ facilitates embryonic development in vitro, promotes blastocyst proliferation and development, and increases blastocyst cell number.^[^ ^29^ ^]^ As for miscarriage, it has also been reported that TGFβ2 are lowly expressed in placental tissues of miscarriage patients relative to healthy controls.^[^ ^30^, ^31^ ^]^ However, the association between TGFβ2 signaling and unexplained miscarriage is still largely elusive. Moreover, the roles of TGFβ2 signaling in trophoblast cell migrasome formation and miscarriage are completely unknown and should be fully investigated.

*Lnc‐HZ05 might regulate TGFβ2 signaling*. LncRNAs (long non‐coding RNAs, >200 nt in length) play crucial roles in numerous cellular processes and various diseases.^[^ ^32^ ^]^ Recently, we have identified a group of novel lncRNAs that regulate trophoblast cell dysfunctions and miscarriage.^[^ ^33^, ^34^, ^35^, ^36^, ^37^, ^38^, ^39^, ^40^, ^41^ ^]^ Among them, a novel lnc‐HZ05 is highly expressed in villous tissues of RM patients relative to their healthy control (HC) and regulates human trophoblast cell proliferation and miscarriage.^[^ ^34^ ^]^ In general, a lncRNA might have multiple functions. Whether lnc‐HZ05 might also regulate TGFβ2 signaling pathway and trophoblast cell migrasome formation remains completely unknown and should be urgently investigated.

*Aim of this study*. In this study, we expect to explore the association, causality, and underlying mechanism among lnc‐HZ05, TGFβ2 signaling, trophoblast cell migrasome formation, and unexplained miscarriage. Based on the assays using human trophoblast cells, RM and HC villous tissues, and placental tissues of a mouse miscarriage model, we find that lnc‐HZ05 specifically suppresses TGFβ2‐promoted trophoblast cell migrasome formation and thus induces miscarriage. This study not only discovers novel regulatory roles of lnc‐HZ05 and TGFβ2 pathway in the pathogenesis of unexplained miscarriage but also provides potential targets for prediction of and treatment against unexplained miscarriage.

## 2 Results

### 2.1 TGFβ2 Signaling‐Regulated Migration/Invasion and Migrasome Formation were Associated with Miscarriage

#### 2.1.1 TGFβ2 Signaling‐Regulated Migration/Invasion and Migrasome Formation were Suppressed In Rm Versus Hc Villous Tissues and were Associated with Miscarriage

*Migration/invasion and migrasome formation were suppressed in RM* versus *HC villous tissues*. To compare the differences and to explore the pathogenesis of unexplained recurrent miscarriage (RM), we collected villous tissue samples from RM patients and their matched healthy control (HC) group (n = 30). The known clinical causes that might induce or cause miscarriage have been excluded. Their characteristic, such as age, body mass index (BMI), gestational days, RBC, WBC, Hb, smoking, and drinking did not show significant differences between these two groups (Table S1, Supporting Information). Two pairs of random HC and RM villous tissues were used for total transcriptome sequencing, giving 717 up‐regulated mRNAs and 765 down‐regulated mRNAs with difference >2‐fold and *p* < 0.05 (**Figure** **1**A). GO analysis of these differentially expressed mRNAs (DEMs) showed that migration/invasion was top significantly altered in RM versus HC villous tissues (Figure 1B). MMP‐2 (matrix metalloproteinase‐2) is a typical protein for migration/invasion.^[^ ^42^, ^43^ ^]^ Cell migration/invasion are always accompanied with migrasome formation. NDST1 (bifunctional heparan sulfate N‐deacetylase/N‐sulfotransferase 1) and TSPAN4 (tetraspanin 4) are two classical indicators for migrasome formation.^[^ ^20^, ^44^, ^45^ ^]^ IHC analysis showed that the protein levels of MMP‐2, NDST1, and TSPAN4 were lower in RM versus HC villous tissues (Figure 1C; Figure S1A, Supporting Information). Moreover, the protein levels of MMP‐2, NDST1 and TSPAN4 were also lower in RM versus HC villous tissues (Figure 1D,E). These results showed that migration/invasion and migrasome formation were suppressed in RM versus HC villous tissues. Multivariate logistic regression analysis by adjusting for all these variables (age, BMI, gestational days, RBC, WBC, Hb, smoking, and drinking) showed that the protein levels of MMP‐2, NDST1, and TSPAN4 were positively associated with miscarriage (Figure 1F). Receiver Operating Characteristic (ROC) curve analysis also showed that their protein levels were associated with miscarriage (Figure 1G). Taken together, these results showed that migration/invasion and migrasome formation were suppressed in RM versus HC villous tissues and the suppressed migration/invasion and migrasome formation were positively associated with miscarriage.

Figure 1TGFβ2 signaling, migration/invasion, and migrasome formation were suppressed in RM vs HC villous tissues and were associated with unexplained miscarriage. A) Volcano plot analysis of the DEMs in RM vs HC villous tissues with difference >2‐fold and *p* < 0.05 (each n = 2), with TGFβ2 shown as purple dot. B) Gene ontology (GO) analysis of the DEMs in RM versus HC villous tissues. C) IHC staining of MMP‐2 and NDST1 protein (shown in brown) in RM and HC villous tissues and their relative quantification. Scale bars, 50 µm. D,E) Western blotting analysis of MMP‐2, NDST1 and TSPAN4 protein levels in RM and HC villous tissues and their relative quantification (each n = 12). F) Multivariate logistic regression analysis of MMP‐2, NDST1, and TSPAN4 protein levels in HC and RM villous tissues by adjusting for all these variables (each n = 12). G) The ROC curve of MMP‐2, NDST1 and TSPAN4 protein with AUC values. H) KEGG analysis of the down‐regulated mRNAs in RM versus HC villous tissues. I) RT‐qPCR analysis of TGFβ2 mRNA levels in HC and RM villous tissues (each n = 30). J) IHC staining of TGFβ2 protein (shown in brown) in HC and RM villous tissues and its relative quantification. Scale bars, 50 µm. K,L) Western blotting analysis of the protein levels of TGFβ2, TGFβR2, Smad3, and pSmad3 in HC and RM villous tissues, and their relative quantification (each n = 12). M) The protein levels of TGFβ2, Smad3, and pSmad3 in Swan 71 cells with TGFβ2 overexpression and their relative quantification. N) The protein levels of TGFβ2, Smad3, and pSmad3 in Swan 71 cells with TGFβ2 knockdown and their relative quantification. O,P) Transwell assay analysis of the migration/invasion of Swan 71 cells with overexpression or knockdown of TGFβ2, and their quantification. Scale bars, 100 µm. Q) The protein levels of NDST1 in Swan 71 or HTR‐8/SVneo cells with TGFβ2 overexpression and its relative quantification. R) Migrasome assays showed the formation of migrasome in Swan 71 cells overexpressing TSPAN4‐GFP and TGFβ2, and their quantification (n = 100). Scale bar, 10 µm. All results are representative data from three independent experiments. Data are presented as mean ± SD. The unpaired two‐tailed Student's *t*‐test C, E, I, J, L, M, Q, and R) and one‐way ANOVA with the Tukey's multiple comparison test (N and P) were used for statistical analysis. *p < 0.05, **p < 0.01, ***p < 0.001, ****p < 0.0001.

**Figure:**
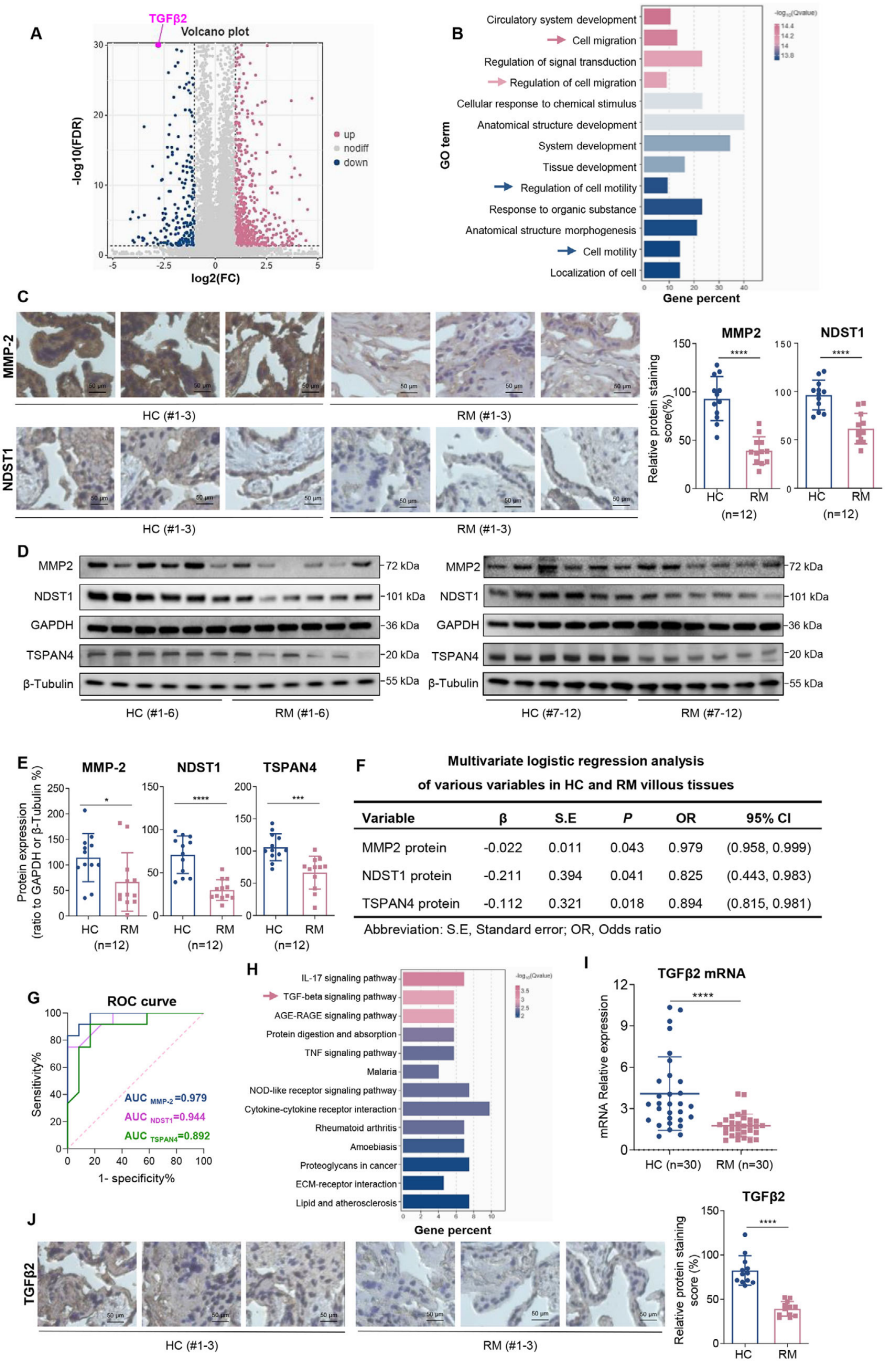

**Figure:**
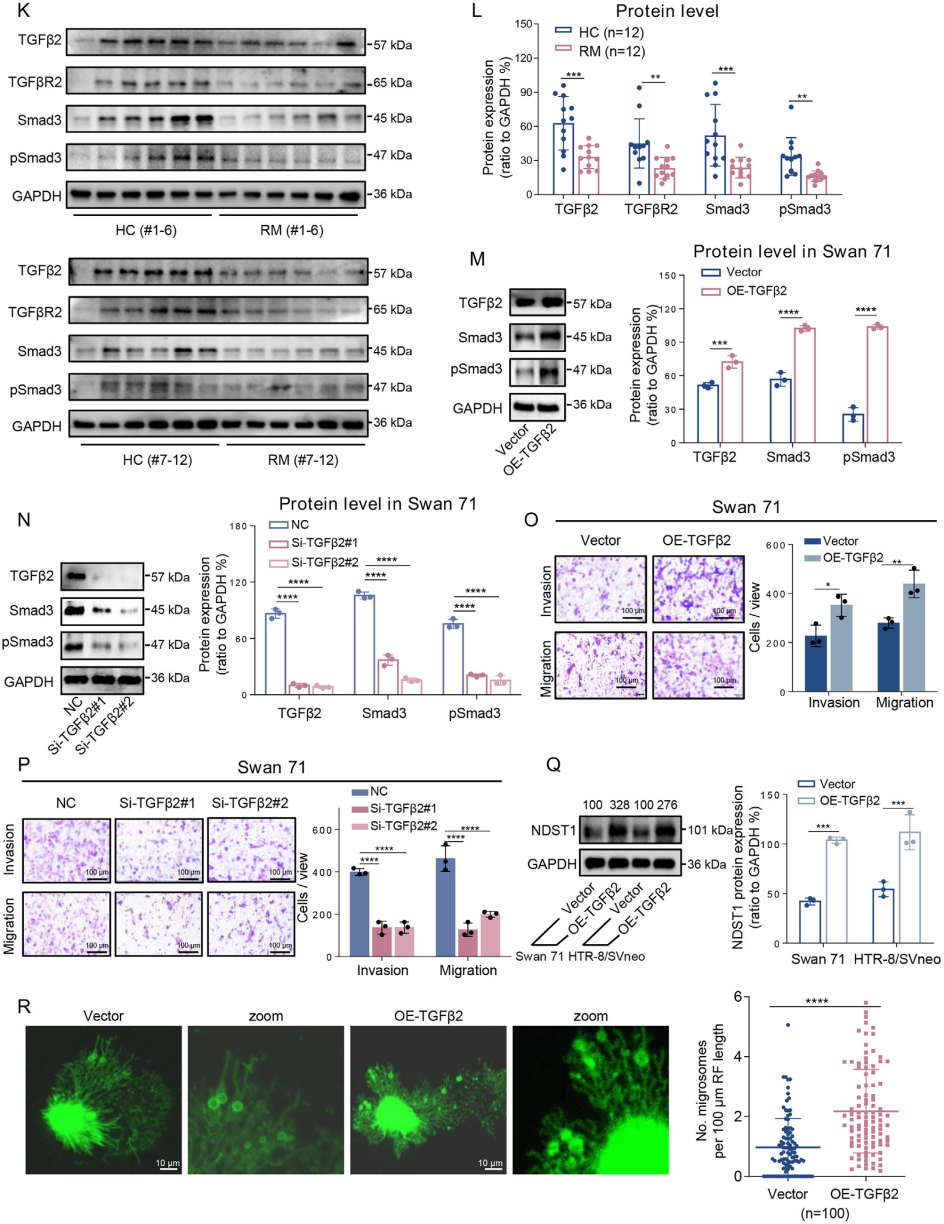

*TGFβ2 signaling was suppressed in RM* versus *HC villous tissues*. Then, we explored which proteins might involve in the suppressed migration/invasion and migrasome formation. Among the differentially expressed mRNAs (DEMs), we identified that TGFβ2 was one of the top significantly down‐regulated mRNAs in RM versus HC villous tissues (Figure 1A). KEGG analysis of these DEMs showed that TGFβ signaling was significantly altered in RM versus HC villous tissues (Figure 1H). In general, TGFβ2/TGFβR2/Smad3 is a typical TGFβ signaling in oral cancer cells and myogenic or chondrogenic progenitor cells.^[^ ^46^, ^47^ ^]^ However, the roles of this signaling in trophoblast cell migrasome formation and miscarriage are still completely unknown. Therefore, in this study, we focused on this TGFβ2/TGFβR2/Smad3 signaling. Experimentally, RT‐qPCR confirmed that the mRNA levels of TGFβ2 were lower in RM versus HC villous tissues (Figure 1I). IHC and Western blot analysis showed that the protein levels of TGFβ2, TGFβR2, Smad3, and p‐Samd3 were all lower in RM versus HC villous tissues (Figure 1J‐L). Multivariate logistic regression analysis by adjusting for all the variables and ROC curve analysis showed that the protein levels of TGFβ2, TGFβR2, Smad3, and p‐Samd3 were all negatively associated with miscarriage (Figure S1B,C, Supporting Information). To further validate this signaling, we overexpressed or silenced TGFβ2 in human trophoblast Swan 71 or HTR‐8/SVneo cells (Figure S1D,E, Supporting Information), which have been widely used as cell models in various miscarriage studies.^[^ ^36^, ^39^, ^40^, ^48^ ^]^ TGFβ2 overexpression up‐regulated, whereas TGFβ2 knockdown down‐regulated, the protein levels of TGFβR2, Smad3, and pSmad3 in Swan 71 or HTR‐8/SVneo cells (Figure 1M,N; Figure S1F,G, Supporting Information). Therefore, the (TGFβ2‐TGFβR2)/Smad3/pSmad3 signaling pathway was suppressed in RM versus HC villous tissues and this suppressed pathway was associated with miscarriage.

*TGFβ2 pathway promoted trophoblast cell migration/invasion and migrasome formation*. It has been reported that TGFβ2 functions for endogenous migration/invasion in pancreatic carcinomas or mammary cells.^[^ ^47^, ^49^ ^]^ To explore its functions in human trophoblast cells, we found that TGFβ2 overexpression promoted, whereas its knockdown suppressed, trophoblast cell migration/invasion (Figure 1O,P; Figure S1H,I, Supporting Information). Moreover, overexpression of TGFβ2 also up‐regulated the protein levels of NDST1 and TSPAN4 (Figure 1Q; Figure S1J, Supporting Information) and promoted the formation of migrasomes (vesicle‐like morphology on the fibers that protrude from the rear of cells, Figure 1R) in human trophoblast cells. Therefore, this TGFβ2 signaling promoted trophoblast cell migration/invasion and migrasome formation in human trophoblast cells.

#### 2.1.2 Tgfβ2 Signaling‐Mediated Migration/Invasion and Migrasome Formation were Suppressed in Placental Tissues of a Mouse Miscarriage Model

**Figure 2:**
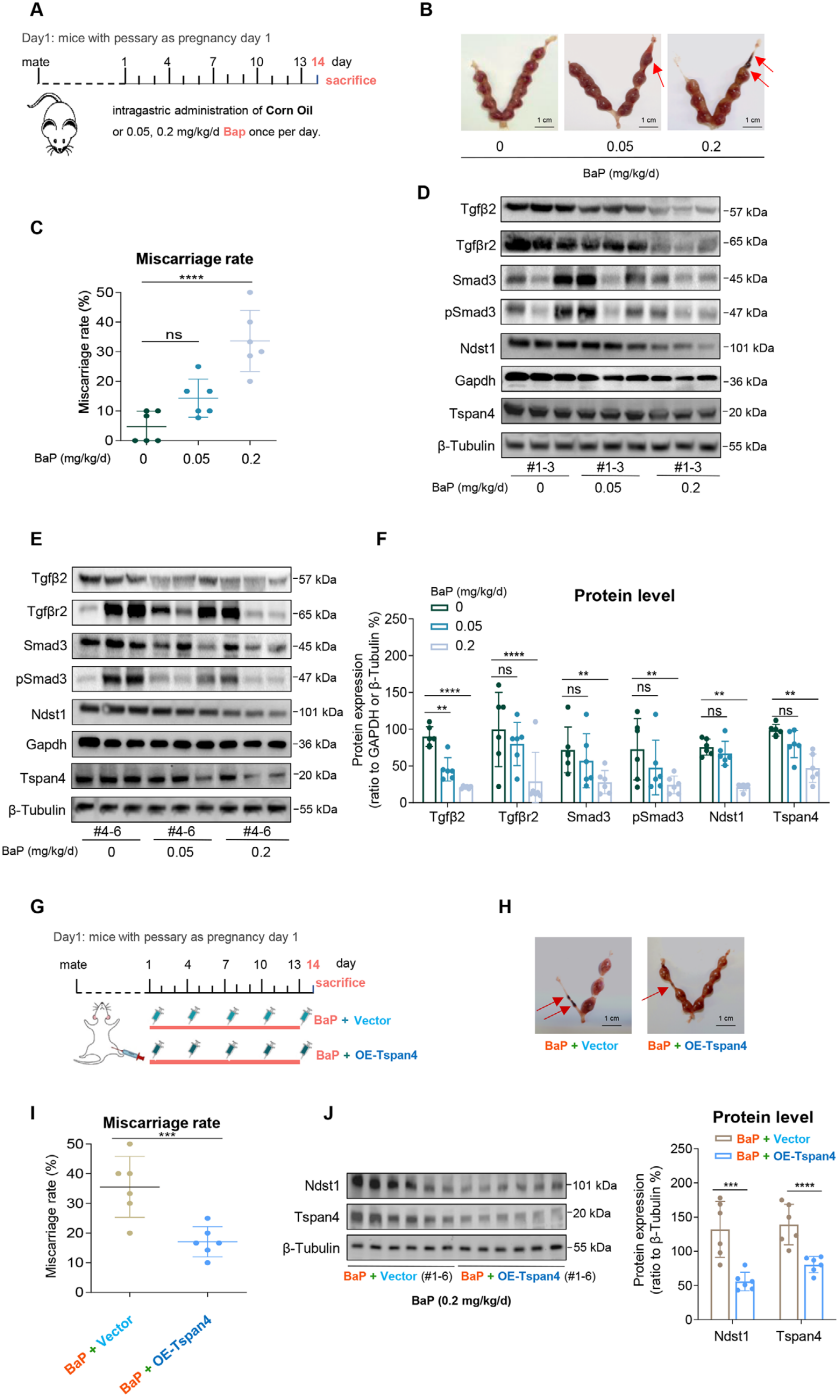
Tgfβ2 signaling, migration/invasion, and migrasome formation were suppressed in placental tissues of a mouse miscarriage model. A) The scheme for BaP‐exposed mouse model with miscarriage. Pregnant mice were daily given 0, 0.05, or 0.2 mg kg^−1^ BaP in corn oil by oral gavage from D1 to D13 and were euthanized on D14 for collection of uterus (each n = 6). B) Embryo resorption (indicated by red arrows) in BaP‐exposed mice (scale bar, 1 cm). C) The average miscarriage rates (the ratios of embryo resorption) in BaP‐exposed mice (each n = 6). D,E,F) The protein levels of murine Tgfβ2, Tgfβr2, Smad3, pSmad3, Ndst1, and Tspan4 in placental tissues of BaP‐exposed mice and their relative quantification (each n = 6). G) The scheme for a mouse miscarriage intervention model. H,I) Embryo resorption (indicated by the red arrows) and miscarriage rates in 0.2 mg kg^−1^ BaP‐exposed mice supplemented with murine Tspan4. J) The protein levels of Ndst1 and Tspan4 in 0.2 mg kg^−1^ BaP‐exposed mice supplemented with Tspan4, with β‐Tubulin as internal standard (each n = 6). All results are representative data from three independent experiments. Data are presented as mean ± SD. The unpaired two‐tailed Student's *t*‐test I,J) and one‐way ANOVA with the Tukey's multiple comparison test C,F). *p < 0.05, **p < 0.01, ***p < 0.001, ****p < 0.0001.

To explore the association among TGFβ2 pathway, migration/invasion, migrasome formation, and miscarriage in vivo, we constructed a BaP‐exposed mouse miscarriage model, which has been widely used in various miscarriage studies.^[^ ^38^, ^41^, ^50^ ^]^ The embryo adsorption was increased and miscarriage rates were elevated in this mouse miscarriage model (**Figure** **2**A–C, n = 6), confirming the successful construction of this model. UCSC‐BLAT analysis showed that TGFβ2, TGFβR2, Smad3, NDST1, and TSPAN4 were all conserved in amino acid sequences among human, mouse, bovine, canis familiaris, and rhesus (Table S8, Supporting Information), indicating that this signaling pathway might have similar functions in both human and mouse. The mRNA levels of murine Tgfβ2 (Figure S2A, Supporting Information) and the protein levels of murine Tgfβ2, Tgfβr2, Smad3, pSmad3, Ndst1, and Tspan4 (Figure 2D–F) were all lower in placental tissues of the miscarriage mice, indicating that this Tgfβ2 pathway, migration/invasion, and migrasome formation were all suppressed in placental tissues of the miscarriaged mice. These results further confirmed that the suppressed TGFβ2 pathway, migration/invasion, and migrasome formation were associated with mouse miscarriage.

#### 2.1.3 Recovery of Migrasome Formation by Supplement with Tspan4 could Efficiently Alleviate Miscarriage in Bap‐Induced Mouse Miscarriage Model

To directly explore whether recovery of migrasome formation might alleviate miscarriage, we constructed a miscarriage intervention model in which a plasmid overexpressing murine Tspan4, with empty vector as control, was intraperitoneally injected into BaP‐exposed pregnant mice once per three days (Figure 2G). In control group, BaP exposure resulted in high miscarriage rate; however, the miscarriage rate was lower in Tspan4‐treated group (n = 6, Figure 2H,I). Analysis of the placental tissues showed that the protein levels of Tspan4 and Ndst1 were higher in the treated groups relative to control group (Figure 2J). Collectively, recovery of migrasome formation by supplement with murine Tspan4 could efficiently alleviate miscarriage in BaP‐induced mouse miscarriage model.

### 2.2 Lnc‐HZ05 Specifically Suppressed TGFβ2‐Mediated Migration/Invasion and Migrasome Formation in Human Trophoblast Cells

#### 2.2.1 TGFβ2 Signaling‐Mediated Migration/Invasion and Migrasome Formation were Suppressed by a lncRNA (lnc‐HZ05) in Trophoblast Cells

**Figure 3:**
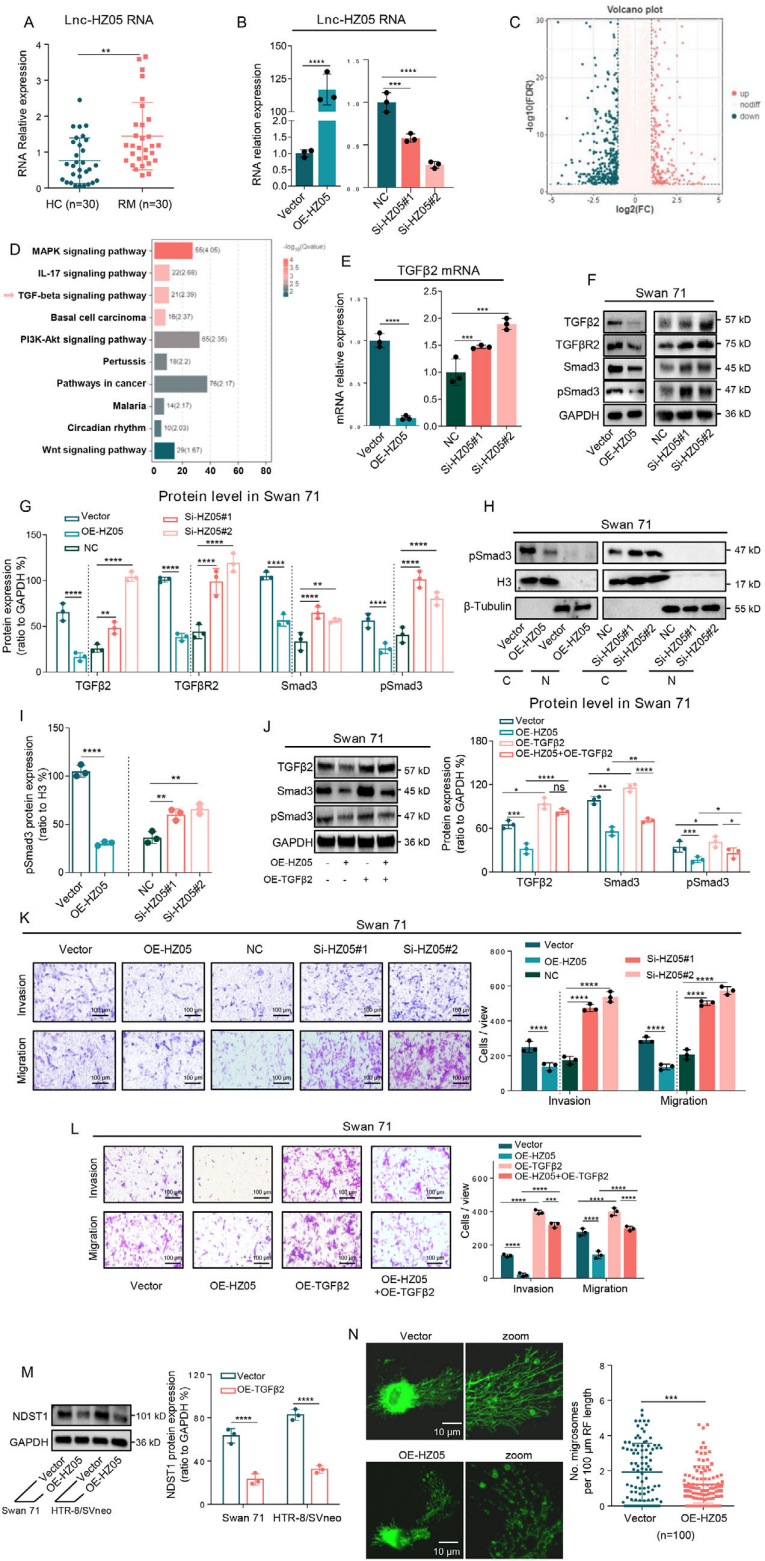
Lnc‐HZ05 down‐regulated TGFβ2 signaling and suppressed trophoblast cell migration/invasion and migrosome formation. A) RT‐qPCR analysis of lnc‐HZ05 expression levels in HC and RM villous tissues (each n = 30). B) Lnc‐HZ05 levels in Swan 71 cells with lnc‐HZ05 overexpression or knockdown. C) Volcano plot analysis of the DEMs in lnc‐HZ05‐overexpressed Swan 71 cells versus control cells with difference >2‐fold and *p* < 0.05. D) KEGG analysis of the down‐regulated mRNAs in lnc‐HZ05‐overexpressed Swan 71 cells vs control cells. E) The mRNA levels of TGFβ2 in Swan 71 cells with lnc‐HZ05 overexpression or knockdown. F,G) The protein levels of TGFβ2, TGFβR2, Smad3, and pSmad3 in Swan 71 cells with lnc‐HZ05 overexpression or knockdown and their relative quantification. H,I) The nuclear cytoplasmic distribution of pSmad3 protein levels in Swan 71 with lnc‐HZ05 overexpression or knockdown, with Tubulin as cytoplasmic (C) indicator and H3 as nuclear (N) indicator, and its relative quantification. J) The protein levels of TGFβ2, Smad3, and pSmad3 in Swan 71 cells with co‐overexpression of lnc‐HZ05 and TGFβ2 and their relative quantification. K) Transwell assay analysis of the migration/invasion of Swan 71 cells with lnc‐HZ05 overexpression or knockdown, and their quantification. Scale bars, 100 µm. L) Transwell assay analysis of the migration/invasion of Swan 71 cells with co‐overexpression of lnc‐HZ05 and TGFβ2 and their relative quantification. Scale bars, 100 µm. M) The protein levels of NDST1 in Swan 71 or HTR/SVneo cells with lnc‐HZ05 overexpression and its relative quantification. N) Migrasome assays showed the formation of migrasome in Swan 71 cells overexpressing TSPAN4‐GFP and lnc‐HZ05, and their quantification (n = 100). Scale bar, 10 µm. All results are representative data from three independent experiments. Data are presented as mean ± SD. The unpaired two‐tailed Student's *t*‐test A, B, E, G, I, K, M, and N) and one‐way ANOVA with the Tukey's multiple comparison test B, E, G, and I–L) were used for statistical analysis. **p < 0.01, ***p < 0.001, ****p < 0.0001.

*Sequencing analysis showed that lnc‐HZ05 might regulate TGFβ2 signaling*. Since lncRNAs play essential roles in regulation human trophoblast cell functions, subsequently, we explored whether lncRNAs might regulate TGFβ2 signaling, migration/invasion, and migrasome formation. In our recent study, we have identified a novel lnc‐HZ05 that was highly expressed in RM versus HC villous tissues and regulated human trophoblast cell proliferation and miscarriage.^[^ ^34^ ^]^ In this study, using the newly collected RM versus HC villous tissues, we further confirmed that lnc‐HZ05 was highly expressed in RM versus HC tissues (n = 30, **Figure** **3**A). To discover the potential regulatory roles of lnc‐HZ05, lnc‐HZ05 was overexpressed in Swan 71 cells (Figure 3B) and these cells, together with empty vector cells as control, were used for mRNA sequencing, giving 711 up‐regulated mRNAs and 755 down‐regulated mRNAs with difference >2‐fold and *p* < 0.05 (Figure 3C). KEGG analysis showed that cell motility was top significantly regulated by lnc‐HZ05 (Figure S3A, Supporting Information). KEGG pathway analysis showed that TGF‐β pathway was top significantly regulated by lnc‐HZ05 (Figure 3D). Therefore, the sequencing data suggested that lnc‐HZ05 might regulate TGFβ2 pathway, migration/invasion, and migrasome formation.

*Lnc‐HZ05 down‐regulated TGFβ2 signaling and suppressed migration/invasion and migrasome formation*. To experimentally validate this, lnc‐HZ05 was overexpressed or silenced in human trophoblast cells (Figure 3B). Overexpression of lnc‐HZ05 down‐regulated, while knockdown of lnc‐HZ05 up‐regulated, the mRNA levels of TGFβ2 (Figure 3E) and the protein levels of (TGFβ2‐TGFβR2)/Smad3/pSmad3 pathway in Swan 71 and HTR‐8/SVneo cells (Figure 3F,G; Figure S3B,C, Supporting Information). In this pathway, pSmad3 functions as a transcription factor in cell nucleus.^[^ ^46^ ^]^ Nucleoplasm distribution assays showed that overexpression of lnc‐HZ05 decreased, whereas lnc‐HZ05 knockdown increased, the nuclear abundance of pSmad3 in trophoblast cells (Figure 3H,I). Co‐transfection assays further showed that the down‐regulation of (TGFβ2‐TGFβR2)/Smad3/pSmad3 pathway caused by lnc‐HZ05 overexpression was reversed by overexpressing TGFβ2 (Figure 3J; Figure S3D, Supporting Information), confirming that lnc‐HZ05 down‐regulated this signaling pathway. As for cell phenotypes, lnc‐HZ05 overexpression suppressed trophoblast cell migration/invasion and lnc‐HZ05 knockdown promoted cell migration/invasion (Figure 3K; Figure S3E, Supporting Information). Similarly, the suppression of migration/invasion caused by lnc‐HZ05 overexpression was reversed by overexpressing TGFβ2 (Figure 3L; Figure S3F, Supporting Information). Meanwhile, lnc‐HZ05 overexpression also down‐regulated the protein levels of NDST1 and TSPAN4 (Figure 3M; Figure S3G, Supporting Information) and suppressed migrasome formation in human trophoblast cells (Figure 3N). Collectively, these results suggested that lnc‐HZ05 suppressed migration/invasion and migrasome formation by down‐regulating this (TGFβ2‐TGFβR2)/Smad3/pSmad3 signaling in human trophoblast cells.

#### 2.2.2 Lnc‐HZ05 Suppressed TGFβ2 mRNA Transcription in Human Trophoblast Cells

**Figure 4:**
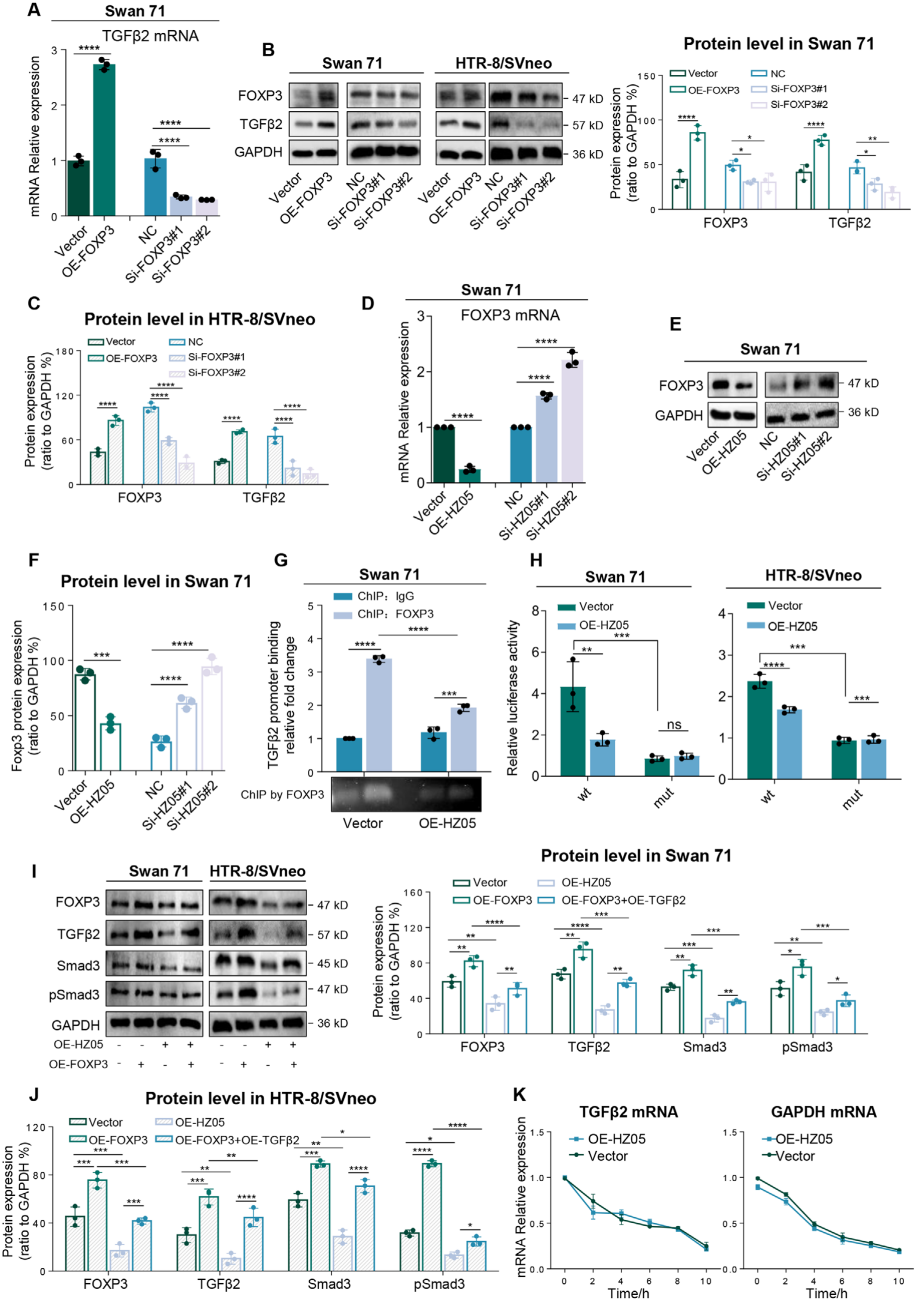
Lnc‐HZ05 suppressed TGFβ2 mRNA transcription in human trophoblast cells. A) The mRNA levels of TGFβ2 in Swan 71 cells with FOXP3 overexpression or knockdown. B,C) The protein levels of TGFβ2 and FOXP3 in Swan 71 or HTR‐8/SVneo cells with FOXP3 overexpression or knockdown and their relative quantification. D–F) The mRNA and protein levels of FOXP3 in Swan 71 cells with lnc‐HZ05 overexpression or knockdown and its relative quantification. G) ChIP assay analysis of the levels of TGFβ2 promoter region enriched by FOXP3 in Swan 71 cells with lnc‐HZ05 overexpression. H) Dual‐luciferase reporter assay analysis of the transcription activity of FOXP3 at wild‐type (wt) or mutant (mut) promoter region of TGFβ2 in Swan 71 or HTR‐8/SVneo cells with lnc‐HZ05 overexpression. I,J) The protein levels of FOXP3, TGFβ2, Smad3, and pSmad3 in Swan 71 or HTR‐8/SVneo cells with co‐overexpression of FOXP3 and lnc‐HZ05 and their relative quantification. K) The mRNA levels of TGFβ2 and GAPDH in 10 µm ActD‐treated Swan 71 cells within 10 h. All results are representative data from three independent experiments. Data are presented as mean ± SD. The unpaired two‐tailed Student's t‐test A–D and F) and one‐way ANOVA with the Tukey's multiple comparison test (A–D, and F–J) were used for statistical analysis. **p < 0.01, ***p < 0.001, ****p < 0.0001.

*Lnc‐HZ05 suppressed FOXP3‐mediated TGFβ2 transcription*. Subsequently, we explored how lnc‐HZ05 down‐regulated TGFβ2 expression levels. First, TGFβ2 transcription was explored in human trophoblast cells. We expected to identify a transcription factor of TGFβ2 that could also be regulated by lnc‐HZ05. Predicted by PROMO, FOXP3 might be a transcription factor of TGFβ2.^[^ ^51^ ^]^ Experimentally, overexpression of FOXP3 up‐regulated TGFβ2 mRNA and protein levels; whereas knockdown of FOXP3 down‐regulated TGFβ2 levels in both trophoblast cells (**Figure** 4 A‐C; Figure S4A,B, Supporting Information). Meanwhile, lnc‐HZ05 overexpression down‐regulated, whereas lnc‐HZ05 knockdown up‐regulated, the mRNA and protein levels of FOXP3 (Figure 4D–F). ChIP assays showed that FOXP3 could bind with the promoter region of TGFβ2, a binding that was weakened with lnc‐HZ05 overexpression (Figure 4G). Dual‐luciferase reporter assays further demonstrated that FOXP3 showed transcription activity using wild‐type (wt) but not mutant (mut) sequence in the promoter region of TGFβ2; however, lnc‐HZ05 overexpression reduced this transcription activity (Figure 4H). Moreover, co‐transfection assays further showed that the down‐regulation of (TGFβ2‐TGFβR2)/Smad3/pSmad3 signaling caused by lnc‐HZ05 overexpression was reversed by co‐overexpressing FOXP3 (Figure 4I,J). However, overexpression or knockdown of lnc‐HZ05 did not affect TGFβ2 mRNA stability (Figure 4K). Taken together, these results showed that FOXP3 was a transcription factor of TGFβ2 and lnc‐HZ05 suppressed FOXP3‐mediated TGFβ2 transcription in human trophoblast cells.

#### 2.2.3 Lnc‐HZ05 Promoted Autophagy Degradation of TGFβ2 Protein in Human Trophoblast Cells

**Figure 5:**
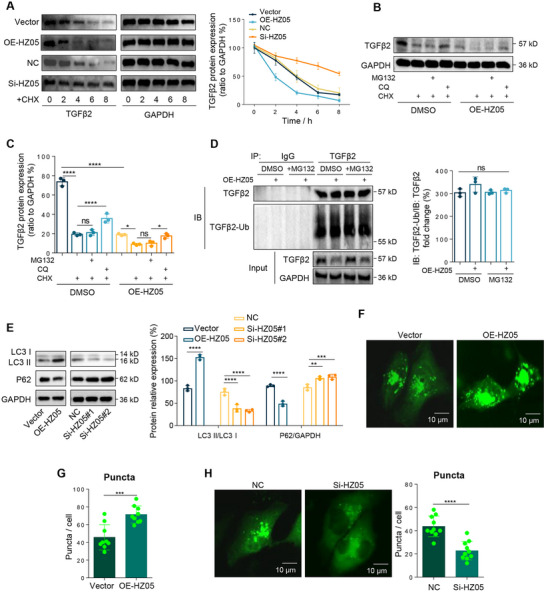
Lnc‐HZ05 promoted TGFβ2 protein autophagy degradation in human trophoblast cells. A) The protein levels of TGFβ2 in 10 µM CHX‐treated Swan 71 cells with lnc‐HZ05 overexpression or knockdown within 8 h and its relative quantification. B,C) The protein levels of TGFβ2 in lnc‐HZ05‐overexpressed and CHX‐treated Swan 71 cells with co‐treatment with 10 µm MG132 or 50 µm CQ and its relative quantification. D) IP assays using identical and limited TGFβ2 antibody showed the levels of poly‐ubiquitinated TGFβ2 (TGFβ2‐Ub) in Swan 71 cells with lnc‐HZ05 overexpression or 10 µm MG132 treatment and its relative quantification, with the protein levels of TGFβ2 in cell lysates. E) The ratios of LC3II/LC3I protein and the protein levels of P62 in Swan 71 cells with lnc‐HZ05 overexpression or knockdown and their relative quantification. F–H) The formation of autophagosomes as indicated by the number of LC3‐GFP puncta (shown in green) in Swan 71 cells with lnc‐HZ05 overexpression or knockdown and their quantification (n = 10). Scale bar: 10 µm. All results are representative data from three independent experiments. Data are presented as mean ± SD. The unpaired two‐tailed Student's t‐test E, G, and H) and one‐way ANOVA with the Tukey's multiple comparison test (C–E) were used for statistical analysis. **p < 0.01, ***p < 0.001, ****p < 0.0001.

*Autophagy degradation of TGFβ2*. Having known that lnc‐HZ05 regulated TGFβ2 at mRNA levels, subsequently, we further explored whether lnc‐HZ05 might also regulate TGFβ2 at protein levels. Overexpression of lnc‐HZ05 reduced TGFβ2 protein stability; whereas knockdown of lnc‐HZ05 enhanced TGFβ2 protein stability (**Figure** **5**A). In general, proteins were degraded through ubiquitination‐proteasome pathway or autophagy‐lysosome pathway, which could be specifically blocked by proteasome inhibitor MG132 or lysosomal inhibitors CQ, respectively.^[^ ^52^, ^53^ ^]^ Treatment of trophoblast cells with CQ increased the accumulation of TGFβ2, whereas treatment with MG132 did not obviously increase its accumulation, either in trophoblast cells or in lnc‐HZ05‐overexpressed trophoblast cells (Figure 5B,C), indicating that TGFβ2 protein was primarily degraded through autophagy pathway. To exclude the possible ubiquitination degradation, the levels of poly‐ubiquitinated TGFβ2 (TGFβ2‐Ub), which was considered as an intermediate for ubiquitination degradation, was analyzed in human trophoblast cells. IP assays showed that TGFβ2 could be ubiquitinated; however, alteration of lnc‐HZ05 did not obviously affect the levels of TGFβ2‐Ub (Figure 5D), suggesting that lnc‐HZ05 might not affect the ubiquitination degradation of TGFβ2 in trophoblast cells.

*Lnc‐HZ05 promoted autophagy degradation of TGFβ2*. To explore whether lnc‐HZ05 might regulate autophagy degradation of TGFβ2, we determined several autophagy protein biomarkers. Overexpression of lnc‐HZ05 increased the ratio of protein levels of LC3II/LC3I and reduced the protein levels of P62; whereas knockdown of lnc‐HZ05 reduced the ratio of protein levels of LC3II/LC3I and increased the protein levels of P62 (Figure 5E), indicating that lnc‐HZ05 promoted autophagy in human trophoblast cells. Moreover, autophagosome formation assays using GFP‐LC3 showed that lnc‐HZ05 overexpression promoted the formation of autophagosomes; whereas lnc‐HZ05 knockdown reduced their formation (Figure 5F–H). Collectively, these results showed that lnc‐HZ05 promoted autophagy and the autophagy degradation of TGFβ2 and thus down‐regulated TGFβ2 protein levels in human trophoblast cells.

#### 2.2.4 Lnc‐HZ05 Impaired TGFβ2/TGFβR2 Protein Interactions in Human Trophoblast Cells

**Figure 6:**
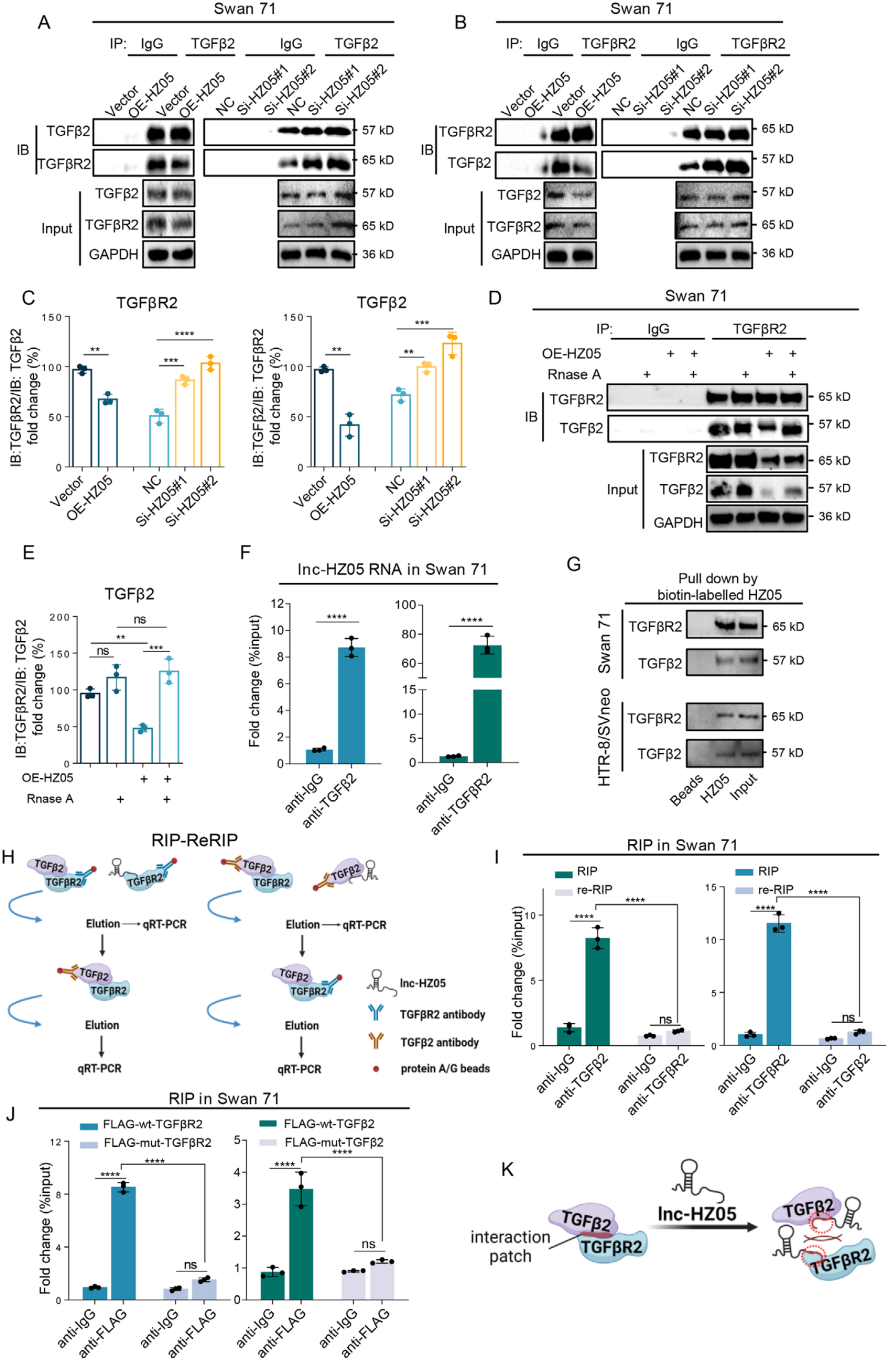
Lnc‐HZ05 impaired TGFβ2/TGFβR2 protein interactions in human trophoblast cells. A–C) IP assays using identical and limited TGFβ2 or TGFβR2 antibody showed the protein levels of the immunoprecipitated TGFβR2 or TGFβ2, respectively, in Swan 71 cells with lnc‐HZ05 overexpression or knockdown and their relative quantification, with the protein levels of TGFβ2 and TGFβR2 in cell lysates. D,E) IP assays using identical and limited TGFβR2 antibody showed the protein levels of the immunoprecipitated TGFβ2 in Swan 71 cells with lnc‐HZ05 overexpression and Rnase A treatment and its relative quantification, with the protein levels of TGFβ2 and TGFβR2 in cell lysates. F) RIP assays using identical and excessive TGFβ2 or TGFβR2 antibody showed that lnc‐HZ05 was pulled down by TGFβ2 or TGFβR2 in Swan 71 cells. G) The protein levels of TGFβ2 or TGFβR2 pulled down by biotin‐labeled lnc‐HZ05 in Swan 71 cells or HTR8/SVneo cells. H) Schematic diagram of RIP‐re‐RIP. In the first round of RIP, one antibody was used to immunoprecipitate proteins containing lnc‐HZ05; in the second round of RIP, the other antibody was used to immunoprecipitate the remaining proteins containing lnc‐HZ05. In both rounds, lnc‐HZ05 were separated and detected. I) The levels of lnc‐HZ05 enriched by TGFβ2 or TGFβR2 in the first and second rounds of RIP‐re‐RIP assays in Swan 71 cells. J) The levels of lnc‐HZ05 pulled down by Flag‐labeled wild‐type (wt) or mutant (mut) TGFβ2 or TGFβR2 in RIP assays. K) The schematic diagram that lnc‐HZ05 impaired the protein interactions between TGFβ2 and TGFβR2. All results are representative data from three independent experiments. Data are presented as mean ± SD. The unpaired two‐tailed Student's *t*‐test (C) and one‐way ANOVA with the Tukey's multiple comparison test C, E, I, and J). ** *p* < 0.01, *** *p* < 0.001, **** *p* < 0.0001.

*Lnc‐HZ05 impaired TGFβ2/TGFβR2 protein interactions*. It has been reported that TGFβ2 and TGFβR2 formed a complex to promote migration/invasion in VBW myofibroblasts cancer cells.^[^ ^54^ ^]^ To validate this in human trophoblast cells, we performed IP assays and found that TGFβ2 and TGFβR2 could be pulled down by each other in both Swan 71 and HTR‐8/SVneo cells (Figure S4C, Supporting Information), indicating the formation of protein complex in trophoblast cells. Subsequently, we evaluated whether lnc‐HZ05 might affect their protein interactions. IP assays using limit but identical target antibody showed that lnc‐HZ05 overexpression weakened their interactions; whereas lnc‐HZ05 knockdown enhanced their interactions (**Figure** **6**A–C; Figure S4D–G, Supporting Information). Immunofluorescence assays also supported that both TGFβ2 and TGFβR2 proteins were co‐localized in trophoblast cells, and this co‐localization was decreased with lnc‐HZ05 overexpression and increased with lnc‐HZ05 knockdown (Figure S4H–J, Supporting Information). To further validate this, cell lysates were treated with Rnase A to completely degrade lnc‐HZ05 without affecting the protein levels of TGFβ2 and TGFβR2 in cell lysates (Figure S4K, Supporting Information). IP assays using limit but identical target antibody showed that this treatment enhanced the binding between TGFβ2 and TGFβR2 (Figure 6D,E, Figure S4L, Supporting Information). Taken together, lnc‐HZ05 impaired the protein interactions between TGFβ2 and TGFβR2 in human trophoblast cells.

*Lnc‐HZ05 interacted with either TGFβ2 or TGFβR2*. Furthermore, we explored how lnc‐HZ05 impaired the binding between TGFβ2 and TGFβR2. First, we determined whether lnc‐HZ05 might interact with TGFβ2 or TGFβR2. RIP assays showed that lnc‐HZ05 could be pulled down by TGFβ2 or TGFβR2 in trophoblast cells (Figure 6F). RNA pulldown assays using biotin‐labeled lnc‐HZ05 also showed that both TGFβ2 and TGFβR2 proteins could be pulled down by lnc‐HZ05 (Figure 6G), indicating that lnc‐HZ05 interacted with TGFβ2 or TGFβR2. Immunofluorescence assays also showed that lnc‐HZ05 was co‐localized with TGFβ2 or TGFβR2 protein in trophoblast cells (Figure S4M,N, Supporting Information). RIP‐re‐RIP assays using one antibody targeting one protein followed by using the other antibody targeting the other protein (Figure 6H) showed that lnc‐HZ05 was pulled down by the first protein but little by the second protein (Figure 6I; Figure S4O, Supporting Information). Therefore, there results showed that a lnc‐HZ05 molecule could interact with either TGFβ2 or TGFβR2 protein but could not simultaneously with both proteins.

*Lnc‐HZ05 interacted with the interaction patch on TGFβ2 or TGFβR2*. It has been reported that TGFβ2 interacts with TGFβR2 through 1‐112 amino acid residues on TGFβ2 and 38‐153 amino acid residues on TGFβR2.^[^ ^55^ ^]^ To explore their protein interactions, we deleted these residues to generate Flag‐labeled mut‐TGFβ2 or mut‐TGFβR2. IP assays using identical but limit Flag antibody showed that mut‐TGFβ2 or mut‐TGFβR2 greatly reduced its protein interactions with TGFβR2 or TGFβ2, respectively (Figure S4P,Q, Supporting Information). Moreover, RIP assays showed that mut‐TGFβ2 or mut‐TGFβR2 also impaired its binding with lnc‐HZ05 (Figure 6J), indicating that lnc‐HZ05 interacted with TGFβ2 or TGFβR2 through this interaction patch. Taken together, lnc‐HZ05 interacted with the specific interaction patch on TGFβ2 or TGFβR2 and thus impair their protein interactions (Figure 6K).

#### 2.2.5 Lnc‐HZ05‐S1 (Segment 1, 1–83 nt) impaired TGFβ2/TGFβR2 Protein Interactions and Suppressed Trophoblast Migration/Invasion and Migrasome Formation

**Figure 7:**
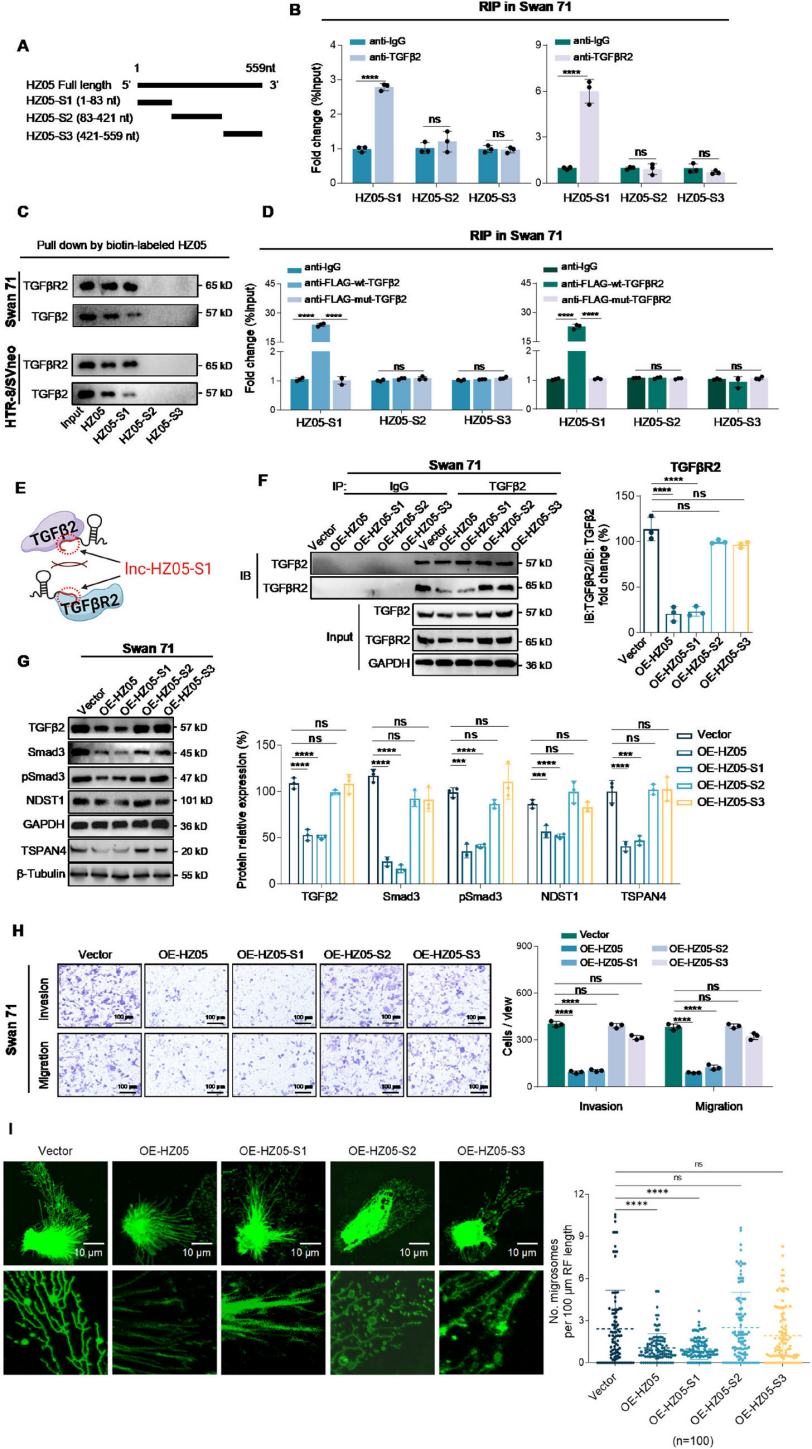
Lnc‐HZ05‐S1 impaired TGFβ2/TGFβR2 protein interactions and also suppressed trophoblast cell migration/invasion. A) Lnc‐HZ05 was divided into three segments: lnc‐HZ05‐S1, lnc‐HZ05‐S2, and lnc‐HZ05‐S3. B) RIP assay analysis of the levels of lnc‐HZ05‐S1, lnc‐HZ05‐S2, and lnc‐HZ05‐S3 that was pulled down by TGFβ2 or TGFβR2 in Swan 71 cells treated with RNase T1. C) RNA pulldown assay analysis of the protein levels of TGFβ2 or TGFβR2 that was pulled down by biotin‐labeled various lnc‐HZ05 in Swan 71 cells or HTR‐8/SVneo cells. D) The levels of lnc‐HZ05‐S1, lnc‐HZ05‐S2, or lnc‐HZ05‐S3 pulled down by Flag‐labeled wild‐type (wt) or mutant (mut) TGFβ2 or TGFβR2 in RIP assays. E) The schematic diagram that lnc‐HZ05‐S1 impaired the protein interactions between TGFβ2 and TGFβR2. F) IP assays using identical but limited TGFβ2 antibody showed the protein levels of the immunoprecipitated TGFβR2 in Swan 71 cells with overexpression of lnc‐HZ05, lnc‐HZ05‐S1, lnc‐HZ05‐S2, or lnc‐HZ05‐S3 and its relative quantification, with the protein levels of TGFβ2 and TGFβR2 in cell lysates. G) The protein levels of TGFβ2, Smad3, pSmad3, NDST1, and TSPAN4 in Swan 71 with overexpression of lnc‐HZ05, lnc‐HZ05‐S1, lnc‐HZ05‐S2, or lnc‐HZ05‐S3, and their relative quantification. H) Transwell assay analysis of the migration/invasion of Swan 71 cells with co‐overexpression of lnc‐HZ05, lnc‐HZ05‐S1, lnc‐HZ05‐S2, or lnc‐HZ05‐S3 and their relative quantification. Scale bars, 100 µm. I) Migrasome assays showed the formation of migrasome in Swan 71 cells with overexpression of lnc‐HZ05, lnc‐HZ05‐S1, lnc‐HZ05‐S2, or lnc‐HZ05‐S3 (n = 100). Scale bar, 10 µm. All results are representative data from three independent experiments. Data are presented as mean ± SD. The one‐way ANOVA with the Tukey's multiple comparison test B, D, F–H). ** *p* < 0.01, *** *p* < 0.001, **** *p* < 0.0001.

*Lnc‐HZ05‐S1 interacted with TGFβ2 or TGFβR2*. To further identify which segment of lnc‐HZ05 might interact with TGFβ2 or TGFβR2, lnc‐HZ05 was truncated into three segments (**Figure** **7**A). RIP‐Rnase T assays showed that only the first segment (HZ05‐S1, 1–83 nt), but not other segments (HZ05‐S2 or HZ05‐S3), was pulled down by TGFβ2 or TGFβR2 (Figure 7B). RNA pull‐down assays using biotin‐labeled lnc‐HZ05 or its various segments also confirmed that TGFβ2 and TGFβR2 could be pulled down by lnc‐HZ05 or HZ05‐S1 but not by other segments (Figure 7C). RIP‐Rnase T assays using Flag‐labeled wt‐ or mut‐TGFβ2 or TGFβR2 further confirmed that only the wt‐proteins, but not mut‐proteins, could pull down HZ05‐S1 but not other segments of lnc‐HZ05 (Figure 7D). Therefore, these data confirmed that the first segment (lnc‐HZ05‐S1, 1–83 nt) in lnc‐HZ05 interacted with the specific interaction patch on TGFβ2 or TGFβR2 in human trophoblast cells (Figure 7E).

*Lnc‐HZ05‐S1 impaired TGFβ2 and TGFβR2 protein interactions*. Meanwhile, we also investigated the effects of each segment of lnc‐HZ05 on protein interactions between TGFβ2 and TGFβR2. IP assays using limit and identical TGFβ2 or TGFβR2 antibody showed that only lnc‐HZ05 or HZ05‐S1, but not other segments, impaired the protein interactions between TGFβ2 and TGFβR2 (Figure 7F; Figure S5A, Supporting Information). Thus, lnc‐HZ05‐S1 might act as a specific adhesion reagent, which specifically adhered to the interaction patch on either protein, and thus impaired their protein interactions (Figure 7E). Collectively, these results showed that the first segment of lnc‐HZ05 (HZ05‐S1) impaired the protein interactions between TGFβ2 and TGFβR2 by directly binding with their specific interaction patch in human trophoblast cells.

*Lnc‐HZ05‐S1 suppressed trophoblast cell migration/invasion and migrasome formation*. Finally, we investigated the effects of each segment of lnc‐HZ05 on its downstream pathway, migration/invasion, and migrasome formation in trophoblast cells. Overexpression of lnc‐HZ05 or HZ05‐S1, but not other segments, down‐regulated the protein levels of TGFβ2, Smad3, pSmad3, NDST1, and TSPAN4 in human trophoblast cells (Figure 7G; Figure S5B,C, Supporting Information). Overexpression of lnc‐HZ05 or HZ05‐S1, but not other segments, suppressed migration/invasion and migrasome formation of human trophoblast cells (Figure 7H,I; Figure S5D, Supporting Information). Taken together, lnc‐HZ05‐S1 (1–83 nt) suppressed trophoblast migration/invasion and migrasome formation, possibly by impairing TGFβ2 and TGFβR2 protein interactions and thus down‐regulating TGFβ2 signaling pathway in human trophoblast cells.

### 2.3 The Upstream of lnc‐HZ05

#### 2.3.1 DNMT1 Suppressed FOXP3‐Mediated Lnc‐HZ05 Transcription

**Figure 8:**
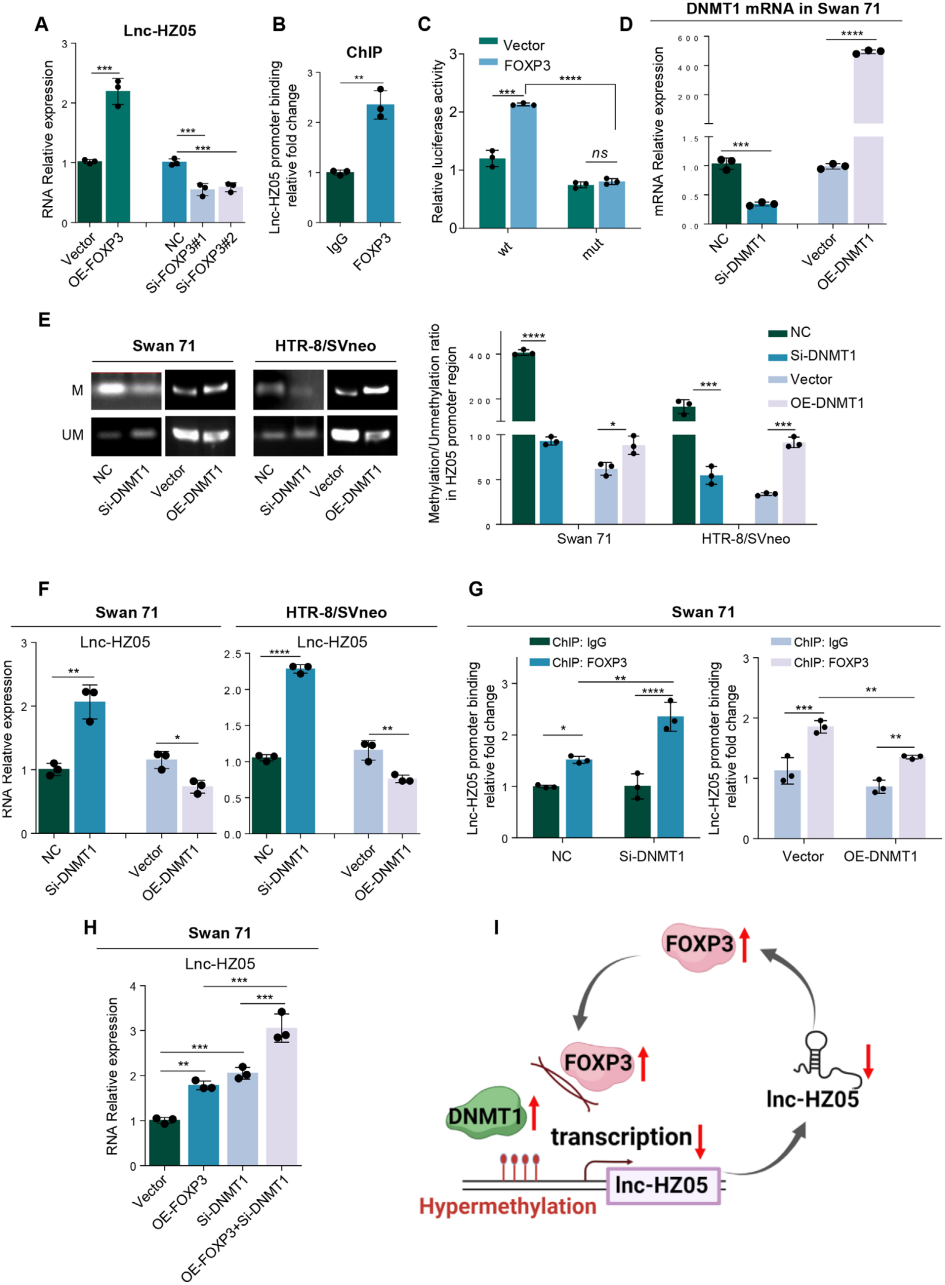
DNMT1 suppressed FOXP3‐mediated lnc‐HZ05 transcription. A) The levels of lnc‐HZ05 in Swan 71 cells with FOXP3 overexpression or knockdown. B) ChIP assay analysis of the levels of lnc‐HZ05 promoter region enriched by FOXP3 in Swan 71 cells. C) Dual‐luciferase reporter assay analysis of the transcription activity of FOXP3 at wild‐type (wt) or mutant (mut) promoter region of lnc‐HZ05 in Swan 71 cells. D) The mRNA levels of DNMT1 in Swan 71 cells with DNMT1 overexpression or knockdown. E) MS‐PCR analysis of the methylation (M) and unmethylation (UM) levels in lnc‐HZ05 promoter region in Swan 71 or HTR8/SVneo cells with DNMT1 overexpression or knockdown and their relative quantification. F) The levels of lnc‐HZ05 in Swan 71 or HTR8/SVneo cells with DNMT1 knockdown or overexpression. G) ChIP assay analysis of the levels of lnc‐HZ05 promoter region enriched by FOXP3 in Swan 71 cells with DNMT1 knockdown or overexpression. H) Lnc‐HZ05 levels in Swan 71 cells with FOXP3 overexpression and DNMT1 knockdown. I) The schematic diagram of a negative feedback loop formed by lnc‐HZ05 and FOXP3. All results are representative data from three independent experiments. Data are presented as mean ± SD. The unpaired two‐tailed Student's *t*‐test (A–F) and one‐way ANOVA with the Tukey's multiple comparison test (A, G, and H) were used for statistical analysis. *p < 0.05, **p < 0.01, ***p < 0.001, ****p < 0.0001.

*FOXP3 was lnc‐HZ05 transcription factor*. Having known the important regulatory roles of lnc‐HZ05 in TGFβ2 signaling pathway, subsequently, we explored what might regulate lnc‐HZ05 expression levels, which have never been investigated in human trophoblast cells. Predicted by PROMO, FOXP3 might also be a transcription factor of lnc‐HZ05. Experimentally, overexpression of FOXP3 up‐regulated, whereas knockdown of FOXP3 down‐regulated, the expression levels of lnc‐HZ05 (**Figure** **8**A). ChIP assays showed that FOXP3 could bind with lnc‐HZ05 promoter region (Figure 8B). Dual‐luciferase reporter assays showed that FOXP3 promoted transcription using wild‐type (wt) but not mutant (mut) sequence in the promoter region of lnc‐HZ05 (Figure 8C). Therefore, these results confirmed that FOXP3 was a transcription factor of lnc‐HZ05 in human trophoblast cells.

*DNMT1 increased DNA methylation levels and suppressed lnc‐HZ05 transcription*. DNA methylation analysis using MethPrimer showed the presence of DNA methylation modification in the promoter region of lnc‐HZ05.^[^ ^56^ ^]^ DNMT1 is a general DNA methylatase in mammalian cells.^[^ ^57^ ^]^ Knockdown of DNMT1 reduced, whereas overexpression of DNMT1 increased, DNA methylation levels in lnc‐HZ05 promoter region (Figure 8D,E; Figure S5E, Supporting Information). As a result, knockdown of DNMT1 up‐regulated, whereas overexpression of DNMT1 down‐regulated, the expression levels of lnc‐HZ05 (Figure 8F). ChIP assays further showed that knockdown of DNMT1 increased, whereas overexpression of DNMT1 reduced, the levels of lnc‐HZ05 promoter region enriched by FOXP3 (Figure 8G). Co‐transfection assays showed that overexpression of FOXP3 promoted lnc‐HZ05 transcription, which was further promoted with co‐knockdown of DNMT1 in human trophoblast cells (Figure 8H). Therefore, DNMT1 increased DNA methylation levels in lnc‐HZ05 promoter region and thus suppressed FOXP3‐mediated lnc‐HZ05 transcription in human trophoblast cells.

*Lnc‐HZ05 and FOXP3 formed a negative feedback loop*. Lnc‐HZ05 down‐regulated FOXP3 expression levels; meanwhile, FOXP3 promoted lnc‐HZ05 transcription. Therefore, both lnc‐HZ05 and FOXP3 formed a negative feedback loop in human trophoblast cells (Figure 8I).

### 2.4 Validation of Cellular Mechanisms in Villous Tissues

#### 2.4.1 Lnc‐HZ05 and TGFβ2 Signaling were Associated in RM Villous Tissues

**Figure 9:**
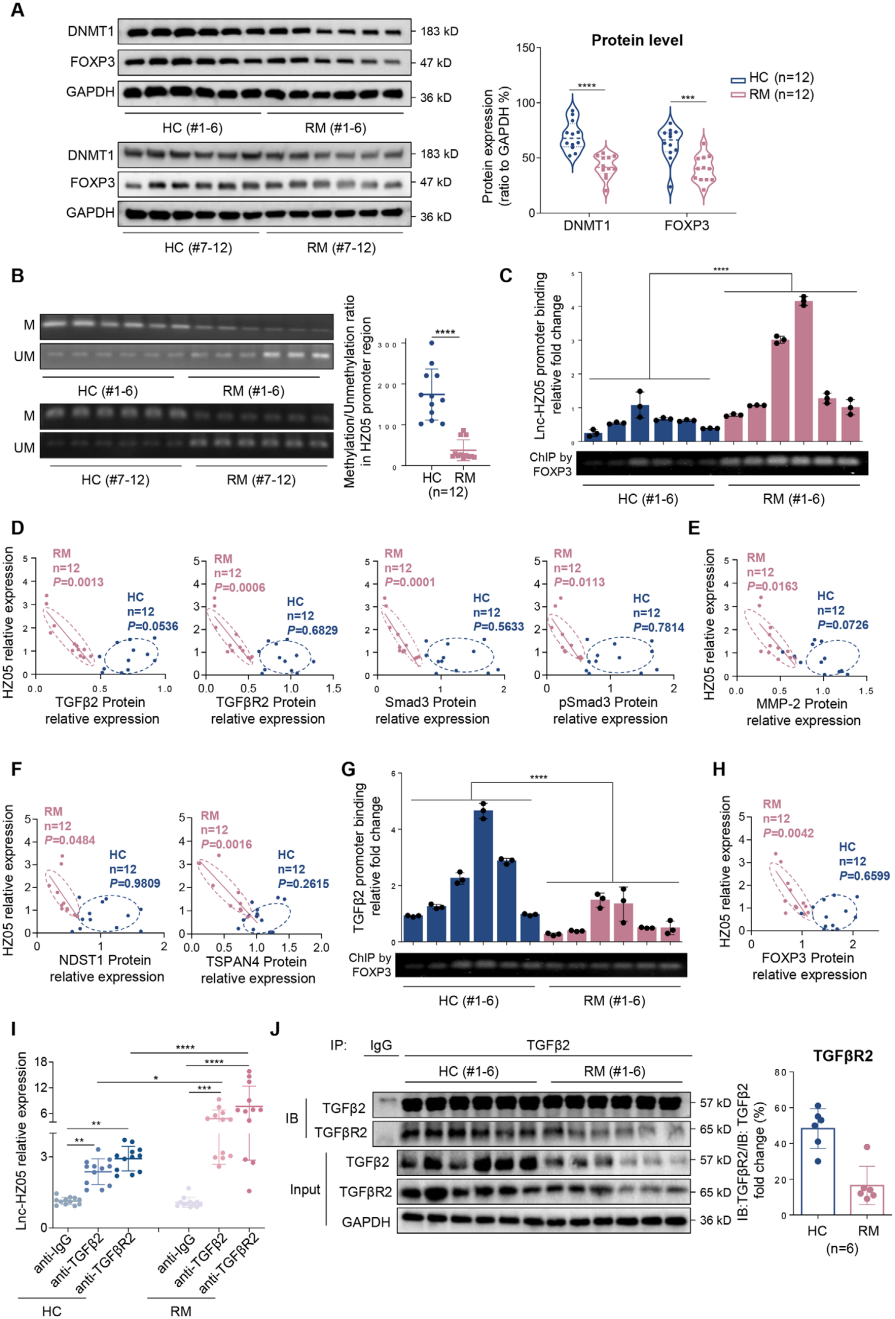
Lnc‐HZ05 and TGFβ2 expression levels in HC and RM villous tissues. A) The protein levels of DNMT1 and FOXP3 in HC and RM villous tissues (n = 12) and their relative quantification. B) MS‐PCR analysis of the methylation (M) and unmethylation (UM) levels in lnc‐HZ05 promoter region in HC and RM villous tissues (n = 12) and its relative quantification. C) ChIP assay analysis of the levels of lnc‐HZ05 promoter region enriched by FOXP3 in HC and RM villous tissues (each n = 6), with IgG as negative control. D–F) The pearson correlation analysis of the relative levels of lnc‐HZ05 with the protein levels of TGFβ2, TGFβR2, Smad3, pSmad3, MMP‐2, NDST1 and TSPAN4 in HC (blue) and RM (pink) groups (n = 12). G) ChIP assay analysis of the levels of TGFβ2 promoter region enriched by FOXP3 in HC and RM villous tissues (each n = 6), with IgG as negative control. H) The pearson correlation analysis of the relative levels of lnc‐HZ05 with that of FOXP3 protein in HC (blue) and RM (pink) groups (each n = 12). I) RIP assay using identical but excessive TGFβ2 or TGFβR2 antibody showed the levels of lnc‐HZ05 enriched by TGFβ2 or TGFβR2 in HC and RM villous tissues (each n = 12). J) IP assay using identical but limited TGFβ2 antibody showed the protein levels of TGFβR2 immunoprecipitated by TGFβ2 in HC and RM villous tissues (each n = 6) and its relative quantification. All results are representative data from three independent experiments. Data are presented as mean ± SD. The unpaired two‐tailed Student's *t*‐test (A, B, and H) and one‐way ANOVA with the Tukey's multiple comparison test (C, E, and G) were used for statistical analysis. Pearson analysis was used for the correlation analysis (D and F). *p < 0.05, **p < 0.01, ***p < 0.001, ****p < 0.0001.

*Lnc‐HZ05 was highly expressed in RM* versus *HC villous tissues*. Since lnc‐HZ05 was highly expressed in RM versus HC villous tissues, the underlying mechanism for its high expression was explored in villous tissues. The protein levels of DNMT1 were lower in RM versus HC villous tissues (**Figure** **9**A), which corresponded lower DNA methylation levels in lnc‐HZ05 promoter region in RM versus HC villous tissues (Figure 9B). Although FOXP3 protein levels were lower in RM versus HC villous tissues (Figure 9A), FOXP3 ChIP assays using identical but excessive anti‐FOXP3 showed that more lnc‐HZ05 promoter region was enriched by FOXP3 in RM versus HC groups (Figure 9C), corresponding to the enhanced lnc‐HZ05 transcription in RM versus HC villous tissues. Collectively, the levels of DNA methylation in lnc‐HZ05 promoter region were lower in RM versus HC villous tissues, which enhanced FOXP3‐mediated lnc‐HZ05 transcription and thus up‐regulated lnc‐HZ05 expression levels in RM versus HC villous tissues.

*Lnc‐HZ05 was negatively associated with TGFβ2 signaling, migration/invasion, and migrasome formation in RM villous tissues*. Pearson correlation analysis showed that the levels of lnc‐HZ05 were negatively and linearly correlated with the protein levels of TGFβ2, TGFβR2, Smad3, or pSmad3 (Figure 9D). Data points in RM and HC groups were relatively separated. Thus, combined with the cellular results, we proposed that lnc‐HZ05 might negatively regulate this TGFβ2 signaling in RM villous tissues. Moreover, the levels of lnc‐HZ05 were also negatively and linearly correlated with the protein levels of MMP‐2, NDST1, or TSPAN4 (Figure 9E,F), indicating that highly expressed lnc‐HZ05 might suppress migration/invasion and migrasome formation in RM villous tissues.

*TGFβ2 pathway was suppressed in RM* versus *HC villous tissues*. The potential mechanism for the down‐regulation of TGFβ2 pathway was also explored in villous tissues. ChIP assays using identical but excessive anti‐FOXP3 showed that less TGFβ2 promoter region was enriched by FOXP3 in RM versus HC groups (Figure 9G). The expression levels of lnc‐HZ05 and FOXP3 were negatively correlated in RM villous tissues (Figure 9H). These results suggested that the up‐regulated lnc‐HZ05 might suppress FOXP3‐mediated TGFβ2 transcription in RM villous tissues. RIP assays using identical but excessive TGFβ2 or TGFβR2 antibody showed that more lnc‐HZ05 was pulled down by either TGFβR2 or TGFβ2 in RM versus HC groups (Figure 9I). IP assays using identical but limited TGFβ2 or TGFβR2 antibody showed that less protein levels of TGFβR2 or TGFβ2 were immunoprecipitated, respectively, in RM versus HC groups (Figure 9J; Figure S5F, Supporting Information), suggesting that the up‐regulated lnc‐HZ05 might impair the protein interactions between TGFβ2 and TGFβR2 in RM villous tissues. Taken together, combined with the cellular results, we proposed that lnc‐HZ05 suppressed FOXP3‐mediated TGFβ2 transcription and also impaired the protein interactions between TGFβ2 and TGFβR2 in RM villous tissues.

### 2.5 Bio‐Targets for Prediction of Miscarriage and Treatment Against Miscarriage

#### 2.5.1 The Levels of TGFβ2 Protein and Lnc‐HZ05 in Serum Predicted Miscarriage

**Figure 10:**
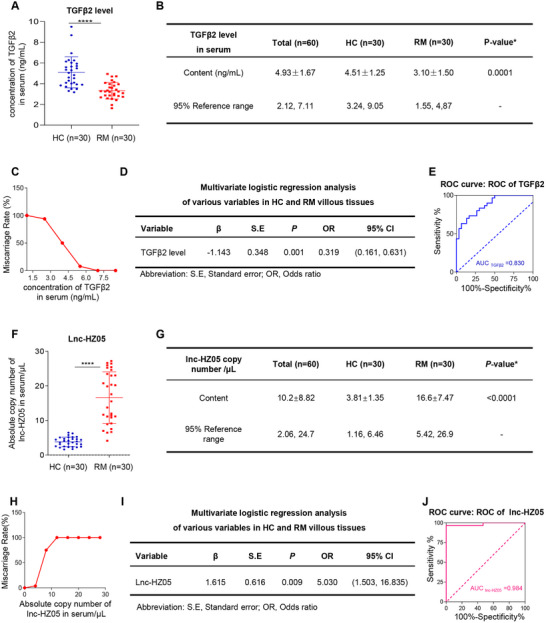
The levels of TGFβ2 protein and lnc‐HZ05 in serum might predict miscarriage risk. A) The levels of TGFβ2 protein in HC and RM serum samples (each n = 30). B) The reference range of TGFβ2 protein levels in HC and RM serum samples (each n = 30). C) The percentage of RM women in total women in each range of serum TGFβ2 protein levels. D) Multivariate logistic regression analysis of TGFβ2 protein levels in HC and RM serum samples by adjusting for all these variables. E) The ROC curve of the serum TGFβ2 protein levels. F) The levels of lnc‐HZ05 absolute copy number in HC and RM serum samples (each n = 30). G) The reference range of lnc‐HZ05 absolute copy number in HC and RM serum samples (each n = 30). H) The percentage of RM women in total women in each range of lnc‐HZ05 absolute copy number. I) Multivariate logistic regression analysis of lnc‐HZ05 absolute copy number in HC and RM serum samples by adjusting for all these variables. J) The ROC curve of lnc‐HZ05 absolute copy number in serum. All results are representative data from three independent experiments. Data are presented as mean ± SD. Unpaired two‐tailed Student's *t*‐test (A and F) were used for statistical analysis. ROC curves were plotted using survival ROC package. ROC AUC (shortly AUC) is calculated as the area under the ROC curve (E and J). *p < 0.05, **p < 0.01, ***p < 0.001, ****p < 0.0001.

*TGFβ2 levels in serum might predict the risk of miscarriage*. TGFβ2, which is a secreted polypeptide growth factor,^[^ ^58^ ^]^ might exist in human serum. Then, we detected its levels in serum and explored whether its serum levels might be used to predict the risk of miscarriage. For this aim, we collected serum samples from unexplained RM and HC women (each n = 30). The levels of TGFβ2 protein were significantly lower in RM versus HC serum samples (**Figure** **10**A). The TGFβ2 protein levels were 4.51 ± 1.25 ng mL^−1^ in HC group and 3.10 ± 1.50 ng mL^−1^ in RM group (Figure 10B). To evaluate the association between serum TGFβ2 protein levels and RM, we divided the protein levels of TGFβ2 into several ranges and counted the numbers of RM and HC women in every range. The proportion of RM women in both HC and RM women showed a decrease trend with increasing serum TGFβ2 protein levels (Figure 10C), indicating that lower protein levels of TGFβ2 in serum were associated with higher risk of miscarriage. This was further confirmed by multivariate logistic regression analysis by adjusting for all these variables (OR = 0.319, 95% CI 0.161‐0.631, Figure 10D). ROC curve analysis showed that the AUC value of serum TGFβ2 protein levels within 95% confidence interval was 0.830, indicating that TGFβ2 might have good predicative capability for miscarriage with high specificity and sensitivity (Figure 10E). Therefore, these data showed that TGFβ2 protein levels in serum might act as a promising detection indicator to predict the risk of miscarriage.

*Lnc‐HZ05 levels in serum might predict the risk of miscarriage*. Subsequently, we also detected whether lnc‐HZ05 in serum might also act as a detection indicator to predict miscarriage risk. Thus, we detected the absolute copy number of lnc‐HZ05 in serum samples. The copies of lnc‐HZ05 were significantly higher in RM versus HC serum samples (Figure 10F). The lnc‐HZ05 levels were 3.81 ± 1.35 copies µL^−1^ in HC group and 16.6 ± 7.47 copies µL^−1^ in RM group (Figure 10G). The percentage of RM women in both HC and RM women in each range of lnc‐HZ05 was increased with increasing lnc‐HZ05 copy numbers (Figure 10H). These results indicated that higher levels of serum lnc‐HZ05 were associated with higher miscarriage rates, which was further confirmed by multivariate logistic regression analysis by adjusting for all these variables (OR = 5.030, 95% CI 1.503–16.835, Figure 10I). ROC curve analysis further showed that the AUC value of lnc‐HZ05 within a 95 % confidence interval was 0.984 (Figure 10J), indicating that lnc‐HZ05 might have good predicative capability for miscarriage with high specificity and sensitivity. Taken together, the data indicated that lnc‐HZ05 in serum could also act as a new detection biomarker to predict the risk for miscarriage.

#### 2.5.2 Treatment Against Miscarriage by Supplementing Murine Tgfβ2 Protein in a Mouse Miscarriage Model

**Figure 11:**
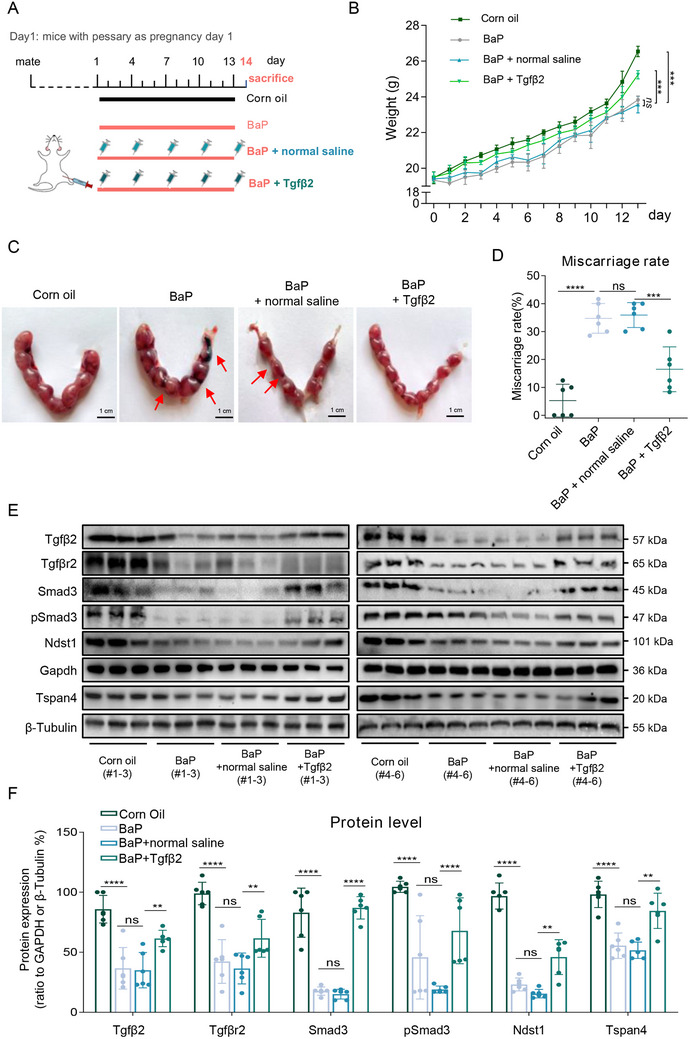
Miscarriage treatment by supplement with murine Tgfβ2 protein in the mouse miscarriage model. A) The scheme for a mouse miscarriage treatment model. Pregnant mice treated with 0 or 0.2 mg kg^−1^ d^−1^ BaP were intraperitoneally injected with saline or recombinant murine Tgfβ2 protein once per three days from D1 to D13 (each n = 6). B) The curves of body weight of 0 or 0.2 mg kg^−1^ d^−1^ BaP‐exposed pregnant mice that were intraperitoneally injected with saline or recombinant murine Tgfβ2 protein once per three days from D1 to D13. C) Embryo resorption (indicated by red arrows) in BaP‐exposed mice with Tgfβ2 supplement (scale bar, 1 cm). D) The average miscarriage rates (embryo resorption ratios) in BaP‐exposed mice with Tgfβ2 supplement (each n = 6). E,F) The protein levels of murine Tgfβ2, Tgfβr2, Smad3, pSmad3, Ndst1, and Tspan4 in placental tissues of BaP‐exposed mice with Tgfβ2 supplement and their relative quantification (each n = 6). All results are representative data from three independent experiments. Data are presented as mean ± SD. The one‐way ANOVA with the Tukey's multiple comparison test (B, D,E). *p < 0.05, **p < 0.01, ***p < 0.001, ****p < 0.0001.

Finally, we explored the potential approach for treatment against miscarriage. The amino acid sequences of TGFβ2, TGFβR2, Smad3, NDST1, and TSPAN4 were all conservative in both human and mouse (Table S8, Supporting Information); however, the analogue of lnc‐HZ05 was not identified in mouse.^[^ ^34^ ^]^ Therefore, we selected recombinant Tgfβ2 protein, which has been widely used in various intervention or treatment in dermal papilla cells, ex vivo cultured human cornea model, lamprey model, or mouse model.^[^ ^58^, ^59^, ^60^, ^61^ ^]^ Then, Tgfβ2 protein, with normal saline as control, was intraperitoneally injected into this mouse miscarriage model (**Figure** **11**A). Since Tgfβ2 is protein, oral administration might result in its digestion and degradation in mouse stomach. Supplement with Tgfβ2 protein could recover the increase in pregnant body weight with pregnancy days, giving similar increase trend as that in the control group (Figure 11B). Moreover, supplement with Tgfβ2 could also efficiently reduce embryo adsorption and reduce miscarriage rates (Figure 11C,D, n = 6). Analysis of the placental tissues further showed that supplement with Tgfβ2 could rescue (i.e., increase) the protein levels of Tgfβ2, Tgfβr2, Smad3, p‐Samd3, Ndst1, and Tspan4 in placental tissues (Figure 11E,F). Therefore, supplement with Tgfβ2 protein did not show obvious adverse effects on the pregnant mice within the tested period and dosage, but could efficiently rescue Tgfβ2 pathway, recover migration/invasion and migrasome formation in placental tissues, and thus alleviate miscarriage in this mouse miscarriage model. These results also validated that supplement with TGFβ2 might be a promising approach for efficient treatment against women miscarriage.

## 3 Discussion

### 3.1 Migrasome Formation is Associated with Miscarriage

As newly identified cellular organelles with vesicle‐like morphology, migrasomes are associated with various diseases, such as angiogenesis, tumor development and immune escape, and proliferative vitreoretinopathy.^[^ ^18^, ^62^, ^63^, ^64^ ^]^ In retinal pigmented epithelium, it has been reported that TGFβ signaling activates Smad3 pathway and triggers TSPAN4 expression and migrasome formation.^[^ ^18^ ^]^ Migrasomes provide specific biochemical information to coordinate organ morphogenesis and play an important role in embryonic development.^[^ ^65^ ^]^ Monocyte migrasomes containing pro‐angiogenic factors could promote capillary formation and monocyte recruitment in chorioallantoic membrane of chick embryos.^[^ ^62^ ^]^ Recently, we have found that polystyrene nanoplastics suppress trophoblast cell migration/invasion and migrasome formation to induce unexplained miscarriage.^[^ ^44^ ^]^ In this study, we find that migrasome formation is suppressed in RM versus HC villous tissues and the suppressed migrasome formation is associated with unexplained recurrent miscarriage. In mouse placental tissues, migrasome formation is also suppressed in the miscarriage group. Moreover, we also identify that lnc‐HZ05 suppresses, whereas TGFβ2 signaling promotes, the formation of migrasomes. Therefore, in this study, we first discover the association among lnc‐HZ05, TGFβ2 signaling, migrasome formation, and miscarriage, revealing novel roles of migrasomes in unexplained recurrent miscarriage.

### 3.2 Regulatory Mechanisms of Lnc‐HZ05

**Figure 12:**
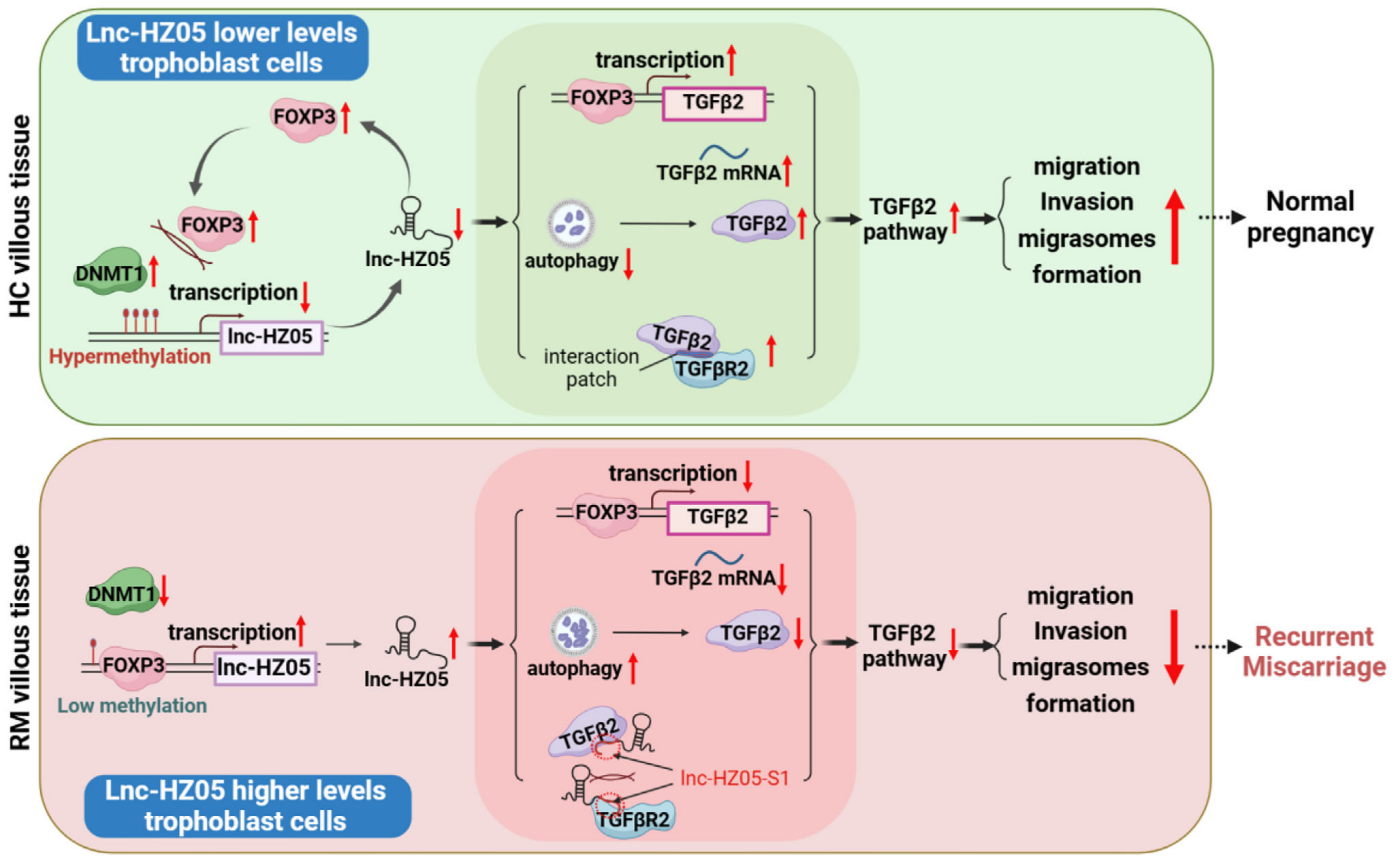
The proposed regulation mechanisms.

The regulatory mechanisms of lnc‐HZ05 are proposed (Figure 12). FOXP3 is a transcription factor of lnc‐HZ05 and promotes its transcription. DNMT1 increases DNA methylation levels in lnc‐HZ05 promoter region and suppresses lnc‐HZ05 transcription. Meanwhile, lnc‐HZ05 also down‐regulates FOXP3 protein levels, and thus both lnc‐HZ05 and FOXP3 form a negative feedback loop. Furthermore, lnc‐HZ05 suppresses FOXP3‐mediated TGFβ2 mRNA transcription and also promotes the autophagy degradation of TGFβ2 protein, both reducing TGFβ2 protein levels. Moreover, the first segment of lnc‐HZ05 (1–83 nt) specifically interacts with the interaction patch on TGFβ2 or TGFβR2 and thus impairs their protein interactions. LncRNA just likes a specific adhesion reagent, which specifically adheres to the interaction patch on either protein, and thus impairs their protein interactions. As far as we known, this is a novel function of lncRNAs that involves in protein‐protein interactions. Therefore, lnc‐HZ05 suppresses (TGFβ2‐TGFβR2)/Smad3/pSmad3 pathway and further suppresses trophoblast cell migration/invasion and migrasome formation. In RM versus HC villous tissues, DNMT1 protein levels are lower, which promotes FOXP3‐mediated lnc‐HZ05 transcription. Subsequently, the up‐regulated lnc‐HZ05 suppresses trophoblast cell migration/invasion and migrasome formation, and further induce miscarriage. Taken together, this study discovers novel epigenetic mechanisms how a lncRNA regulates TGFβ2 signaling, enriching the upstream regulatory mechanism of TGFβ2, a potential target for treatment against miscarriage.

### 3.3 Supplement with TGFβ2 Could Efficiently Treat Against Miscarriage

TGFβ family regulates numerous cellular processes and is associated with multiple diseases.^[^ ^15^, ^25^, ^66^ ^]^ It has been reported that supplement of recombinant murine Tgfβ2 protein significantly improves glucose tolerance and insulin sensitivity in HFD‐induced obese mice.^[^ ^58^ ^]^ Recombinant Tgfβ2 protein could ameliorate the abnormal expression of pro‐inflammatory markers in adipose tissue in mice.^[^ ^67^ ^]^ Some studies have shown that TGFβ2 plays important roles in cell proliferation, invasion/migration, differentiation, decidualization, and placentation.^[^ ^30^, ^68^ ^]^ TGFβ2 expression is regulated by histone H3K4‐specific demethylase KDM5C, and its levels is lower in RM versus HC trophoblastic and decidual cells.^[^ ^31^ ^]^ As a downstream target of miR‐193b‐3p, TGFβ2 promotes HTR‐8/SVneo cell migration and invasion.^[^ ^69^ ^]^ However, the association and causation between TGFβ2 and miscarriage remain largely elusive. Moreover, whether TGFβ2 might be used for miscarriage treatment has never been explored. In this study, we find that TGFβ2 signaling is suppressed in RM versus HC villous tissues and in placental tissues of a mouse miscarriage model. Functionally, TGFβ2 is essential for migration/invasion and migrasome formation, as well as for a healthy pregnancy. In mouse miscarriage model, murine (Tgfβ2‐Tgfβr2)/Smad3/p‐Samd3 signaling in placental tissues are down‐regulated. Supplement with recombinant murine Tgfβ2 protein could efficiently recover Tgfβ2 pathway, restore migration/invasion and migrasome formation in mouse placenta, and further alleviate mouse miscarriage, providing an efficient approach for treatment against miscarriage. In a broader view, supplement with TGFβ2 might also be used for treatment against other placenta‐originated adverse pregnancy outcomes, such as eclampsia, intrauterine growth restriction, or premature birth. However, it still needs to optimize the types of TGFβ2 molecule (such as protein, mRNA, or its overexpression plasmid), delivery methods (such as nanoparticle encapsulation for slow release), delivery approaches (such as oral, injection, vaginal, or systemic administration), and long‐term safety to promote clinical translation.

### 3.4 The Serum Levels of TGFβ2 and Lnc‐HZ05 Might Predict Miscarriage Risk

In addition to miscarriage treatment, TGFβ2 could also be used as detection indicator for various diseases, such as autoimmune diseases, cancer, fibrosis, and cardiovascular diseases.^[^ ^70^, ^71^ ^]^ In this study, we also find that TGFβ2 protein levels in serum are significantly lower in RM versus HC groups and could be used as a detection indicator for prediction of miscarriage risk. Meanwhile, we also find that lnc‐HZ05 levels in serum are significantly higher in RM versus HC groups and could also be used as a detection indicator for prediction of miscarriage risk. In a broader view, both lnc‐HZ05 and TGFβ2 might be used for early diagnose and prediction indicators of other placenta‐originated adverse pregnancy outcomes, which should be further concerned.

### 3.5 LncRNAs have Different Conservation in Human and Mouse

Compared with mRNAs, lncRNAs lack high conservation in sequence and secondary structures among different species.^[^ ^72^, ^73^ ^]^ First, some lncRNA sequences are homologous and their functions are also similar in human and mouse, such as LINC00116,^[^ ^74^ ^]^ lncRNA Ppp1r1b,^[^ ^75^ ^]^ lnc‐HZ06 (causes ferroptosis to induce miscarriage under hypoxia and up‐regulates IL1B and suppresses migration/invasion to induce miscarriage under normoxia),^[^ ^35^, ^36^ ^]^ and lnc‐HZ11 (suppresses EGR1/NF‐κB/CXCL12 pathway and migration/invasion to induce miscarriage.^[^ ^39^ ^]^ Second, some lncRNA sequences are homologous, but their functions are obviously different. For example, lncRNA FAST differs in subcellular localization in human and mouse embryonic stem cells, which leads to their functional divergence in the context of pluripotency regulation.^[^ ^76^ ^]^ In our recent study, we have found that lnc‐HZ08 sequence is conserved in both human and mouse but lnc‐HZ08 could bind with CBL and PI3K,^[^ ^77^ ^]^ or regulate homologous recombination repair of DNA double strand break in human system but could not in mouse system.^[^ ^48^ ^]^ Thirdly, some lncRNAs are present in only human but not in mouse. For example, we have recently found that lnc‐HZ09 suppresses PLD1/RAC1/CDC42 pathway and migration/invasion to induce women miscarriage;^[^ ^38^ ^]^ lnc‐HZ10 suppresses homologous recombination repair to induce miscarriage;^[^ ^48^ ^]^ and lnc‐HZ12 suppresses autophagy degradation of BBC3 to cause apoptosis and induces miscarriage.^[^ ^40^ ^]^ In this study, lnc‐HZ05 suppresses trophoblast cell migrasome formation by disrupting TGFβ2 pathway and induces miscarriage. All these lncRNAs are specifically present in human and mammals and but not in mouse system, demonstrating the specific regulatory roles of these lncRNAs in human trophoblast cell functions and women miscarriage, which should be concerned in drug development and disease therapy. Other mammals models, such as swine or rhesus model, should be used to further explore the roles of mammal specific lncRNAs in the occurrence of miscarriage as well as in the treatment against miscarriage.

### 3.6 Limitations and Prospects

In the future, a larger cohort containing more RM and HC women should be enrolled to provide stronger statistical results and more robust conclusions. In addition to migration/invasion and TGFβ2 signaling, the mRNA sequencing data also suggest that other cell phenotypes and other key pathways might also be regulated, which should also be considered in the future. Since migrasomes could emit cellular components (such as damaged mitochondria), whether these components might induce dysfunctions of other adjacent healthy cells to induce miscarriage should be fully addressed. Since many other lncRNAs are also differentially expressed, the epigenetic regulatory network between TGFβ2 signaling and other lncRNAs should be further studied. In addition to trophoblast cell lines, the roles of lnc‐HZ05 and TGFβ2 signaling might be further explored using primary trophoblast cells and trophoblast organoids, which might be more promising to elucidate the functions of trophoblast cells in women placental tissues. In this study, we focus on miscarriage and did not consider offspring. The roles of lnc‐HZ05 and TGFβ2 signaling in offspring growth, organ development, and multi‐generational epigenetic effects should be further explored. Moreover, optimization of TGFβ2 types, delivery methods, delivery approaches, and long‐term safety should be further considered before clinical translation. Furthermore, the potential reasons for the up‐regulation of lnc‐HZ05 are still largely unclear. Increasing evidence has shown that environmental toxicants might be potential reasons to alter epigenetic regulation and to induce miscarriage. New emerging environmental toxicants should be considered for the occurrence of unexplained miscarriage and other adverse pregnant outcomes.

## 4 Conclusion

In this study, we find that TGFβ2 signaling‐mediated migration/invasion and migrasome formation are suppressed in RM versus HC villous tissues and are negatively associated with unexplained RM. In mechanism, TGFβ2 promotes trophoblast cell migration/invasion and migrasome formation, all of which are suppressed by lnc‐HZ05. In details, lnc‐HZ05 suppresses FOXP3‐mediated TGFβ2 mRNA transcription, promotes autophagy degradation of TGFβ2 protein, impairs TGFβ2/TGFβR2 protein interactions, and eventually induce unexplained miscarriage. Meanwhile, FOXP3 promotes lnc‐HZ05 transcription, thus forming a negative regulatory loop with lnc‐HZ05. The serum levels of TGFβ2 protein are lower and that of lnc‐HZ05 are higher in RM versus HC group and could predict miscarriage risk. In a mouse miscarriage model, supplement with murine Tgfβ2 protein promotes migration/invasion and migrasome formation in placenta and efficiently alleviate mouse miscarriage. This study not only discovers novel biological mechanisms of lnc‐HZ05 and TGFβ2 signaling in the pathogenesis of unexplained miscarriage but also provides potential targets for prediction of and treatment against unexplained miscarriage.

## 5 Experimental Section

### Materials

Cyclohexide (CHX, Cat. No. M4879) and MG‐132 (Cat. No. M1902) were purchased from AbMole Bioscience (Houston, Texas, USA). BaP (purity, 96%, Sigma‐Aldrich) was dissolved in corn oil (Sigma‐Aldrich). All other chemicals were of molecular biology grade. Recombinant murine Tgfβ2 protein (HY‐P70649) was from MedChemExpress (New Jersey, USA).

### Cell Culture

Both Swan 71 and HTR‐8/SVneo cells were developed from the first trimester placental tissues. Swan 71 cells were immortalized by human telomerase; and HTR‐8/SVneo cells were immortalized by SVneo virus. Both trophoblast cell lines had similar migration and invasion ability and the expression levels of biomarkers of extravillous trophoblasts (EVT) and also had similar cell functions and characteristic as fresh primary trophoblast cells isolated from women placental tissues.^[^ ^78^, ^79^ ^]^ Therefore, both Swan 71 and HTR‐8/SVneo cells have been widely used as cell model for miscarriage studies.^[^ ^33^, ^34^, ^35^, ^36^, ^37^, ^38^, ^39^, ^40^, ^41^ ^]^ Both Swan 71 cells and HTR‐8/SVneo cells (within 15 generations) were commercially available (ThermoFisher Scientific, Waltham, MA, USA). Swan 71 cells were calculated in DMEM/F12 medium (Gibco, Invitrogen, Carlsbad, CA, USA) and HTR‐8/SVneo cells were in RPMI 1640 medium (Gibco, Invitrogen, Carlsbad, CA, USA) at 37 °C in a humidified atmosphere containing 5% CO_2_. Both media were supplemented with 10% fetal bovine serum (Gibco), 100 U/mL penicillin, and 100 U/mL streptomycin (Gibco).

### Tissue Collection and Statement

Human villous tissues were collected from 30 patients with unexplained recurrent miscarriage (RM group) and 30 women who sought induced miscarriage to terminate their unwanted pregnancies, which were considered as healthy control (HC) group, at 6–10 weeks of gestation, as the methods described previously.^[^ ^38^, ^41^, ^50^ ^]^ They were all in 25–30 years old. Any woman in both groups with one of the following characteristics was excluded, including abnormal karyotype, abnormal uterus and cervix, abnormal hormone secretion, autoimmune deficiency, endocrine and metabolic diseases, viral infectious diseases, et al., as described previously.^[^ ^38^, ^41^, ^50^ ^]^ All HC group had previous successful pregnancies. HC and RM women did not receive any treatment. The characteristic of these HC and RM women were listed in Table S1 (Supporting Information), including baseline characteristic (age, body mass index), clinical information (gestational days, RBC, WBC, Hb), and lifestyle (smoking, drinking) in the period of three month before miscarriage operation. All these information was obtained from medical records. The villous tissue samples were dissected from other abortive materials, serially washed, immediately frozen in liquid nitrogen, and then stored at −80 °C until RNA extraction and protein separation. Peripheral blood samples were collected in BD Vacutainer SST™ tubes. Serum samples were isolated within 30 min by centrifugation and were stored in aliquot at −80 °C until further use. The experiment protocols have been authorized by the Eighth Affiliated Hospital, Sun Yat‐sen University (Approval no. 2022‐017‐01). Written informed consent has been signed before the study.

### High‐Throughput Transcriptome Sequencing and Data Processing

Two pairs of random HC and RM villous tissues (30 mg) were collected for high‐throughput mRNA sequencing. Swan 71 cells (5 × 10^6^ cells) that were overexposed with lnc‐HZ05, with empty vector cells as control, were also used for high‐throughput mRNA sequencing. Total RNAs were extracted by Trizol reagent (Thermo Fisher Scientific, Waltham, MA, USA) and quantified using NanoDrop2000. RNA quality was assessed by gel electrophoresis and Qubit (Thermo, Waltham, MA, USA). The process included the removal of rRNA, synthesis of double‐stranded cDNA, end repair, degradation of one strand, and enrichment of the other strand by PCR. Strand‐specific libraries were constructed using the TruSeq RNA Sample Preparation Kit (Illumina, San Diego, CA, USA). Sequencing was performed on the Illumina Novaseq 6000 instrument by the commercial service of BGI Genomics Co. Ltd. (Shenzhen, China). Gene expression was calculated and expressed as fragments per kilobase of transcript per million mapped reads (FPKM). The differentially expressed mRNAs (DEMs) with differences >2‐fold and *p* < 0.05 were generated from read counts using the online bioinformatic platform Dr. Tom provided by BGI (biosys.bgi.com/). These DEMs were searched in NCBI database (Gene Bank, Homo sapiens, GRCh38.p14) to determine their genome loci. Subsequently, these DEMs were used for GO (Gene Onotology) analysis (http://geneontology.org/) to generate GO plots and for KEGG (Kyoto Encyclopedia of Genes and Genomes) analysis to generate KEGG pathway plots.

### Cell Transfection

SiRNAs and overexpression plasmids were designed and synthesized by Sangon Biotech Company (Shanghai, China). Swan 71 or HTR‐8/SVneo cells (1 × 10^6^ cells per well) were seeded into six‐well plates for 24 h and then transfected with 5 nM siRNA, 1000 ng pcDNA3.1 overexpression plasmid, or its corresponding negative control in Lipofectamine 3000 (L300015, Invitrogen) for 24 h, and then cells were harvested for analysis. Sequences of siRNAs and their negative control (NC) were shown in Table S2 (Supporting Information). The sequences of genes in overexpression plasmid were listed in Table S3 (Supporting Information). The transfection efficiencies were validated by RT‐qPCR.

### Western Blotting

Human trophoblast cells, women villous tissues, or female placental tissues were lysed with RIPA lysis buffer (Thermo Fisher Scientific) for 30 min on ice followed by high‐speed centrifugation at 13 000 rpm for 15 min. The supernatant was collected and protein concentration was analyzed using Pierce BCA Protein Assay Kit (Pierce, Rockford, IL, USA). Proteins (10–30 µg per well, equal amounts within group but different amounts among groups for better comparison) were separated via SDS‐PAGE and then transferred onto polyvinylidene fluoride (PVDF) membranes (GE Healthcare, United Kingdom). Next, the PVDF membranes were incubated with 5% BSA, followed incubated with primary and secondary antibodies. Primary antibodies included anti‐TGFβ2 (sc‐374659, 1:500, SANTA), anti‐TGFβR2 (sc‐17799, 1:500, SANTA), anti‐Smad3 (5G11, 1:1000, Invitrogen), anti‐pSmad3 (ab52903, 1:1000, Abcam), anti‐FOXP3 (150D/E4, 1:1000, Invitrogen), anti‐NDST1 (sc‐100790,1:500, SANTA), anti‐TSPAN4 (PA5‐69344, 1:1000, Invitrogen), anti‐β‐Actin (ab8226, 1:1000, Abcam), anti‐GAPDH (ab8245, 1:1000, Abcam), and anti‐H3 (ab1791,1:1000, Abcam). Secondary antibodies included HRP‐conjugated anti‐mouse IgG (ab205719, 1:100 000, Abcam) and HRP‐conjugated anti‐rabbit IgG (ab150077, 1:100 000, Abcam). The intensity of each band was quantified by Image J. The value of each band density in experimental and control groups was normalized to that of its corresponding GAPDH band (loading control, ratio to GAPDH%).

### Reverse Transcription and Quantitative Real‐Time PCR (RT‐qPCR)

Total RNAs were extracted from cells or villous tissues with Trizol Reagent (Invitrogen). The first strand cDNA synthesis kit (Takara, Kyoto, Japan) was used for synthesis of cDNA. The isolated RNAs (800 ng) were converted into cDNAs using the first‐strand cDNA synthesis kit (Invitrogen) using the first strand cDNA synthesis kit (Invitrogen). Quantitative real‐time PCR was performed using an iQ5 real‐time detection system (Bio‐Rad Laboratories) and SYBRVR Premix Ex TaqTM II (Bio‐Rad Laboratories). The expression levels of RNAs were analyzed using the 2^−ΔΔCt^ method. GAPDH mRNA was used as the normalization internal control for lncRNAs and mRNAs. The primer sequences are listed in Table S4 (Supporting Information).

### Immunoprecipitation (IP) Assays

Trophoblast cells or women villous tissues were lysed in IP lysis buffer containing protease inhibitors. The lysates were centrifugated at 14 000 × g for 15 min. The supernatant was incubated with the indicated antibody (using identical but limited amount of antibody relative to target protein in cell lysates) coupled on Protein A/G agarose beads (B23201, Bimake) at 4 °C overnight. Beads were washed with IP lysis buffer thrice and were boiled in 50 µL 2× sodium dodecyl sulfate (SDS) loading buffer. Proteins were then analyzed by Western blotting with indicated antibodies.

### Migration/Invasion Assays

For migration assays, trophoblast cells were seeded in the upper transwell chambers (Corning, Lowell, MA, USA). DMEM or RPMI 1640 medium with 10% (v/v) FBS was used in the lower chambers. Then, the cells were fixed by 4% paraformaldehyde and stained with 0.5% crystal violet. The migrated cells were counted from six random high‐power fields on a light microscope. For invasion assays, matrigel (BD Biosciences, Franklin Lakes, NJ, USA) diluted in DMEM/F12 medium (dilution ratio 1:30) were coated on the 24‐well transwell chambers and were solidified at 37 °C for 1 h. Cells were added to the top of matrigel matrix and cultured for 24 h. Afterward, the invaded cells on membranes were counted from six random high‐power fields on a light microscope. For better comparison, equal amounts of cells were used within group but different amounts of cells among groups.

### Protein Stability Assays

Trophoblast cells were treated with 100 µg mL^−1^ cycloheximide (M4879, AbMole) to block protein translation. After incubation for 0, 2, 4, 6, 8, or 10 h, cells were lysed in RIPA buffer to generate whole cell lysates. The remaining TGFβ2 protein levels were detected by Western blotting, with GAPDH protein as internal standard.

### Chromatin Immunoprecipitation (ChIP) Assays

ChIP assays were performed using EZ‐Magna ChIP Chromatin Immunoprecipitation Kit (Cat. No. 17‐408, Millipore). Trophoblast ells were cross‐linked using 1% formaldehyde at 25 °C for 20 min and lysed in SDS buffer. Chromatin was pelleted, re‐suspended in immunoprecipitation buffer, and then sonicated into fragments with 200–1000 bp as determined by 1% agarose gel. Then, the mixture was incubated with FOXP3 or IgG antibody attached on beads at 4 °C overnight. After separation of these beads, DNA bound onto beads were extracted and analyzed by qPCR. The qPCR products were also analyzed by 1% agarose gel. The primer sequences were listed in Table S5 (Supporting Information).

### RNA Immunoprecipitation (RIP) Assays

RIP assays were performed using RNA Immunoprecipitation Kit (Millipore, Burlington, MA, USA). Briefly, trophoblast cell lysates were incubated with magnetic beads conjugated with antibodies against IgG (anti‐IgG), TGFβ2 (anti‐TGFβ2), or TGFβR2 (anti‐TGFβR2) overnight at 4 °C. RNAs that were enriched by proteins on beads were extracted and analyzed by RT‐qPCR. The primer sequences were listed in Table S4 (Supporting Information).

### Biotinylated RNA Pulldown Assays

Biotinylated RNA pulldown assays were performed as described previously.^[^ ^80^ ^]^ Briefly, oligonucleotides of lnc‐HZ05 or its various segments were designed, synthesized, and in vitro transcribed from pGEM‐T‐lnc‐HZ05 or its various segments (pGEM‐T‐lnc‐HZ05‐S1, ‐S2, or ‐S3) (Addgene, sequences in Table S6, Supporting Information). All the transcripts were biotin‐labeled by T7 RNA polymerase using Biotin RNA Labeling Mix (Roche, Basel, Switzerland). After treatment with DNase I, the RNAs were purified with RNeasy Mini Kit (Qiagen, Valencia, CA, USA). The transcripts were then incubated with trophoblastic cells lysates at 4 °C overnight. Finally, the protein‐RNA complexes were separated from streptavidin magnetic beads and the proteins were analyzed by Western blotting using anti‐TGFβ2 or anti‐TGFβR2 antibody.

### Nucleoplasm Distribution Assays

Nucleus and cytoplasm were separated using nuclear/cytoplasmic fractionation Kit (ab289882, abcam). Briefly, Swan 71 or HTR‐8/SVneo cells were lysed in cold cytoplasmic lysis buffer with a homogenizer for 10 min on ice. The released nuclei were collected by centrifugation at 15 000 *g* for 15 min at 4 °C. The supernatant was collected as cytoplasmic proteins. The remained nuclei were then lysed in cold nuclei lysis buffer and vortexed for 10 min for 4 times on ice, and were then centrifuged at 15 000 *g* for 15 min at 4 °C. The supernatant was collected as nuclear proteins. Then, the cytoplasmic and nuclear proteins were analyzed by Western blotting, with tubulin as cytoplasmic marker and H3 as nuclear marker.

### Dual‐Luciferase Reporter Assays

Wild‐type (wt) or mutant (mut) promoter sequence of TGFβ2 or lnc‐HZ05 was fused into the luciferase pmirGLO‐basic reporter vector (Promega, Madison, USA). Swan 71 cells or HTR‐8/SVneo cells were co‐transfected with 100 ng pmirGLO‐wt/mut TGFβ2 or pmirGLO‐wt/mut HZ05 and 200 nM plasmid overexpressing FOXP3 or empty vector in Lipofectamine 3000 (Invitrogen) according to the manufacturer's instructions. The firefly luciferase activity was measured using Dual‐Luciferase Reporter Assay System (Promega) according to the manufacturer's protocols. Briefly, trophoblast cells were incubated for 24 h and lysed in passive lysis buffer on ice for 20 min. Cell lysates were then mixed with luciferase assay reagent. The firefly luciferase activity was measured on a fluorescence analyzer (PE envision, PE, China). Subsequently, cell lysates were incubated with stop reagent and the renilla luciferases activity was measured as background value.

### Methylation‐Specific PCR (MS‐PCR) Assays

Genome DNAs were extracted from trophoblast cells or villous tissues using QIAampDNA Mini Kit (QIAGEN, Dusseldorf, Germany). Genomic DNAs were modified with bisulfite using the EZDNA methylation Kit (Los Angeles, USA) and were immediately used or stored at −80 °C. DNAs were treated with sodium bisulfite, which converted the unmethylated cytosine to uracil, without changes in the methylated cytosine. Then, DNAs were amplified with methylated‐ or unmethylated‐specific primers. The methylated regions were identified using UCSC (https://genome.ucsc.edu/). The primers were designed using MethPrimer (http://www.urogene.org/methprimer). Primer sequences were listed in Table S7 (Supporting Information). Each methylation‐specific PCR reaction contained 100 ng bisulfite‐treated DNA as template, 2 µL each primer, 25 µL Red Taq Polymerase (Sigma‐Aldrich, St. Louis, MO) in a final reaction volume of 50 µL. The program was 95 °C for 5 min and 35 cycles of 94 °C for 20 s, 60 °C for 30 s, and 72 °C for 20 s. PCR products were analyzed with non‐denatured 1% polyacrylamide gel electrophoresis and imaged with ChemiDoc imaging system (Bio‐Rad, Hercules, CA, USA).

### A mouse Miscarriage Model

A mouse miscarriage model was constructed as the method described previously.^[^ ^36^, ^37^, ^39^, ^41^ ^]^ Briefly, pregnant C57BL/6 mice (Charles River Company, Beijing, China) were randomly assigned to three groups (each n = 6), and each group received 0, 0.05, or 0.2 mg kg^−1^ d^−1^ BaP by oral gavage from day 1 (D1) to D13 to give a mouse miscarriage model. To construct a Tspan4 supplement model, pregnant mice were randomly divided into two groups (each n = 6): (1) BaP+Vector group, (2) BaP+OE‐Tspan4 group. From D1 to D13, four groups received corn oil or 0.2 mg kg^−1^ BaP daily by oral gavage. Meanwhile, mice were intraperitoneally injected with 20 µg kg^−1^ pcDNA3.1‐Tspan4 or pcDNA3.1 vector once per three days from D1 to D13. To construct a miscarriage treatment model, pregnant mice were randomly divided into four groups (each n = 6): (1) corn oil group, (2) BaP exposure group, (3) BaP + normal saline group, (4) BaP + Tgfβ2 protein group. From D1 to D13, four groups received corn oil or 0.2 mg kg^−1^ d^−1^ BaP by oral gavage. Meanwhile, mice were also intraperitoneally injected with 0.1 µg kg^−1^ Tgfβ2 protein or normal saline every day from D1 to D13. On D14, all mice were euthanized with nembutal (100 mg kg^−1^) to collect uterus. Miscarriage rate in each mouse was calculated as the number of embryo resorption divided by the number of total embryos. RNAs or proteins were extracted from a randomly selected placenta from each mouse in every group. The animal protocols have been authorized by Medical Ethics Committee of Medical Research Center at the Eighth Affiliated Hospital, Sun Yat‐sen University (Approval no. 2023‐021‐01).

### Statistical Analysis

Every experiment was performed thrice independently with similar results and the data were expressed as mean ± SD (standard deviation, n = 3). Statistical analysis was performed using one‐way analysis of variance with SPSS v24.0 software. The significance of differences between two groups were determined using LSD *t*‐test. Dunnett's or LSD test was used to examine the statistical difference among three or more groups. The correlation analysis of the relative expression levels was performed using Pearson analysis. Receiver Operating Characteristic (ROC) curves were plotted using survival ROC package. Area Under Curve (AUC) was calculated as the area under the ROC curve. Multivariate logistic regression analysis by adjusting for all these variables (age, BMI, gestational days, RBC, WBC, Hb, smoking, and drinking) was used to analyze the odds ratio (OR) of each variable and the risk of unexplained miscarriage and its 95% confidence interval (95% CI). The differences with *p < 0.05 or **p < 0.01 were considered statistically significant and were manifested in each panel.

## Conflict of Interest

The authors declare no conflict of interest.

## Author Contributions

W.C., Y.Z., and Z.Z. are co‐authors and contributed equally to this work. W.C., Y.Z., Z.Z., D.Z., and H.Z. designed the study; W.C., Y.Z., and Z.Z. performed most of the experiments; W.C., Y.Z. Z.Z., D.Z., and H.Z. wrote the draft manuscript; Y.Y., Y.S., and W.H. for the rest experiments; G.G., T.Z., Z.Z., and Z.H. contributed for data analysis; L.Z., Q.S., and R.H. contributed for clinical sample collection.

## Supporting information



Supporting Information

## Data Availability

The data that support the findings of this study are available from the corresponding author upon reasonable request.
