# Supplementary material for: Lnc‐HZ05 Suppresses Trophoblast Cell Migrasome Formation by Disrupting TGFβ2 Pathway in Unexplained Recurrent Miscarriage
Source: Adv Sci (Weinh). 2025 Oct 30;13(2):e17558. doi: 10.1002/advs.202417558 (PMC12786376; doi:10.1002/advs.202417558)
Supplement: Supplementary file 1 — Supporting Information [file ADVS-13-e17558-s001.docx]

**Supplementary Materials**

**Table S1. The characteristic of these HC and RM women.**

| **Characteristic** | | **RM^e^ (n=30)** | **HC^f^ (n=50)** | **P-value** |
| --- | --- | --- | --- | --- |
| Age | | 29.12 ± 1.83^g^ | 28.96 ± 1.92 | 0.510^h^ |
| BMI^a^ | | 25.33 ± 0.63 | 24.66 ± 0.93 | 0.326 |
| Gestational days (d) | | 50 ± 5.11 | 49 ± 4.25 | 0.368 |
| RBC^b^ (10^12^/L) | | 4.53 ± 0.39 | 4.58 ± 0.56 | 0.751 |
| WBC^c^ (10^9^/L) | | 6.65 ± 1.92 | 6.86 ± 1.57 | 0.658 |
| Hb^d^ (g/L) | | 137 ± 15.42 | 140 ± 15.72 | 0.557 |
| Smoking | no | 16 | 18 | 0.668 |
|  | 1-4 times per week | 6 | 5 |  |
|  | > 4 times per week | 8 | 7 |  |
| Drinking | no | 25 | 26 | 0.426 |
|  | 1-4 times per week | 3 | 3 |  |
|  | > 4 times per week | 2 | 1 |  |

^a^BMI: Body Mass Index.

^b^RBC: red blood cell.

^c^WBC: white blood cell.

^d^Hb: Hemoglobin.

^e^RM: Unexplained recurrent miscarriage group.

^f^HC: healthy control group.

^g^mean ± standard deviation (n = 30 in each RM or HC group).

^h^Chi-square test.

**Table S2. Sequences of siRNAs for cell transfection.**

| **Name** | **Sense (5'-3')** | **Antisense (5'-3')** |
| --- | --- | --- |
| si1-HZ05 | CUGUCUAAGAUUGCUGGAUAUTT | AUAUCCAGCAAUCUUAGACAGTT |
| si2-HZ05 | CCUUUCAAGUGACAUUCCUAUTT | AUAGGAAUGUCACUUGAAAGGTT |
| si1-TGFβ2 | UCAAUCUGUCUUUCUCUUGUCTT | CAAGAGAAAGACAGAUUGAGATT |
| si2-TGFβ2 | AAGUAGAAAUAAAUUUAAGAGTT | CUUAAAUUUAUUUCUACUUAATT |
| si1-FOXP3 | UGAAGUAGUCCAUGUUGUGGATT | CACAACAUGGACUACUUCAAGTT |
| si2-FOXP3 | GCACAUUCCCAGAGUUCCUTT | AGGAACUCUGGGAAUGUGCTT |
| si-DNMT1 | UGUUAAGCUGUCUCUUUCCAATT | GGAAAGAGACAGCUUAACAGATT |
| siRNA NC | UUCUCCGAACGUGUCACGUTT | ACGUGACACGUUCGGAGAATT |

**Table S3. DNA sequences used for construction of pcDNA3.1 overexpression plasmids.**

| **Gene** | **Sequence region (5'-3')** | |
| --- | --- | --- |
| LncHZ05 | Full sequence, chr6: 26,193,311-26,193,869, NC-000006.12 | |
| Lnc-HZ05-S1 | CTTCTATACC…CATTGTTTAC, chr6: 26,193,311 - 26,193,394, 1~83 nt | |
| Lnc-HZ05-S2 | CGTATATCAC…ATTCTTCATA, chr6: 26,193,394 - 26,193,732, 83~421 nt | |
| Lnc-HZ05-S3 | ATGAGCTTCA…TAGTATGGCA, chr6: 26,193,732 - 26,193,869, 421~559 nt | |
| TGFβ2 | [NM_001135599.4](https://www.ncbi.nlm.nih.gov/nuccore/NM_001135599.4) |  |
| FOXP3 | NM_001114377.2 |  |
| Tspan4 | NM_001252588.2 |  |

**Table S4. Sequences of the primers used in various PCR assays.**

| **Species** | **Gene** | **Forward (5'-3')** | **Reverse (5'-3')** |
| --- | --- | --- | --- |
| Human | Lnc-HZ05 | CGGAAGAATACCCACCGAGG | TCGATGTTCCTCTACCCCGT |
|  | FOXP3 | GTGGCCCGGATGTGAGAAG | GGAGCCCTTGTCGGATGATG |
|  | TGFβ2 | CAGCACACTCGATATGGACCA | CCTCGGGCTCAGGATAGTCT |
|  | DNMT1 | AGAACGGTGCTCATGCTTACA | CTCTACGGGCTTCACTTCTTG |
|  | GAPDH | TGTGTCCGTCGTGGATCTGA | GCAGCTGTGACACACAGTA |
|  | Lnc-HZ05-S1 (1-83 nt) | CTATACCAGACTCTCAGTATG | AAGAAGTCACCAAACTTAGT |
|  | Lnc-HZ05-S2 (83-421 nt) | ACTGACACTTCAGTTATTCCC | GAATATTTCACCCAGAGCAAT |
|  | Lnc-HZ05-S3 (421-559 nt) | CTTTCTCTCATCCCTTTGTGCT | CCATGCTAAGATTAGTCTTAAG |
|  | Lnc-HZ05 | CGGAAGAATACCCACCGAGG | TCGATGTTCCTCTACCCCGT |
|  | FOXP3 | GTGGCCCGGATGTGAGAAG | GGAGCCCTTGTCGGATGATG |
| Mouse | Tgfβ2 | TCGACATGGATCAGTTTATGCG | CCCTGGTACTGTTGTAGATGGA |
|  | Gapdh | AGGTCGGTGTGAACGGATTTG | AGGTCGGTGTGAACGGATTTG |

**Table S5. Sequences of the primers used in various ChIP PCR assays.**

| **Name** | **Forward primer (5'-3')** | **Reverse primer (5'-3')** |
| --- | --- | --- |
| Lnc-HZ05 | CCGTAGCCGTTCCAGTTCAT | TAGCCGCCAACACCTACTTG |
| TGFβ2 | TGGGATGGCAACCTGCTAAG | TCTCTGAACCACGTGTCTGC |
| GAPDH | TGTGTCCGTCGTGGATCTGA | GCAGCTGTGACACACAGTA |

**Table S6. DNA sequences used for construction of pGEM-T** **plasmids.**

| **Gene** | **Sequence region (5'-3')** |
| --- | --- |
| Lnc-HZ05 | Full sequence, chr6: 26,193,311-26,193,869, NC-000006.12 |
| Lnc-HZ05-S1 | CTTCTATACC…CATTGTTTAC, chr6: 26,193,311 - 26,193,394, 1~83 nt |
| Lnc-HZ05-S2 | CGTATATCAC…ATTCTTCATA, chr6: 26,193,394 - 26,193,732, 83~421 nt |
| Lnc-HZ05-S3 | ATGAGCTTCA…TAGTATGGCA, chr6: 26,193,732 - 26,193,869, 421~559 nt |

**Table S7. Sequences of the primers used in methylation-specific PCR assays.**

| **Gene** | **Forward (5'-3')** | **Reverse (5'-3')** |
| --- | --- | --- |
| M lnc-HZ10 | AAAGATTGAGTTGGGAATTAATTAG | ACTTAAAAAATAAACCAAAAATCAC |
| UM lnc-HZ10 | AGATTAAGTTAGGAATTAATTAAGT | ACTTAAAAAATAAACTAAAAATTAT |

**Table S8. The homology analysis of amino acid sequence of FOXP3, TGFβ2, TGFβR2, Smad3, NDST1, and TSPAN4.**

| **Genes** | **Percent Identity** | | | | | | |
| --- | --- | --- | --- | --- | --- | --- | --- |
|  | Mouse | Pig | Rat | Elephant | Bovine | Goat | Rhesus macaque |
| FOXP3 | 86.9% | 86.8% | 85.8% | 89.2% | 90.0% | 89.8% | 98.6% |
| TGFβ2 | 96.1% | 99.3% | 95.4% | 99.3% | 98.3% | 98.0% | 99.1% |
| TGFβR2 | 92.2% | 87.5% | 91.5% | 93.8% | 91.9% | 91.5% | 98.9% |
| Smad3 | 100.0% | 100.0% | 100.0% | 100.0% | 99.8% | 100.0% | 99.6% |
| NDST1 | 97.8% | 95.6% | 98.0% | 97.7% | 98.2% | 98.4% | 98.3% |
| TSPAN4 | 93.7% | 92.5% | 93.3% | 93.6% | 93.3% | 92.4% | 99.2% |

**Figure S1.**

A	IHC staining of TSPAN4 protein in HC and RM villous tissues and its relative quantification. Scale bars, 50 μm.

B	Multivariate logistic regression analysis of the protein levels of TGFβ2, TGFβR2, Smad3, and pSmad3 in HC and RM villous tissues (n = 12) by adjusting for all these variables.

C	The ROC curves of the protein levels of TGFβ2, TGFβR2, Smad3, and pSmad3.

D, E The mRNA levels of TGFβ2 in Swan 71 or HTR8/SVneo cells with TGFβ2 overexpression or knockdown.

F, G The protein levels of TGFβ2, Smad3, and pSmad3 in HTR-8/SVneo cells with TGFβ2 overexpression or knockdown.

H, I	Transwell assay analysis of the migration/invasion of HTR-8/SVneo cells with TGFβ2 overexpression or knockdown and their relative quantification. Scale bars, 100 μm.

J	The protein levels of TSPAN4 in Swan 71 or HTR-8/SVneo cells with TGFβ2 overexpression and its relative quantification.

All results are representative data from three independent experiments. Data are presented as mean ± SD. The unpaired two-tailed Student’s t-test (A, D, E, G, I ,and J) and one-way ANOVA with the Tukey’s multiple comparison test (D, E, G, I) were used for statistical analysis. **P* < 0.05, ***P* < 0.01, ****P* < 0.001, *****P* < 0.0001.

**Figure S2.**

A	The mRNA levels of Tgfβ2 in placental tissues of mice exposed to 0 (with corn oil as solvent), 0.05, or 0.2 mg/kg/d BaP (each n = 6).

All results are representative data from three independent experiments. Data are presented as mean ± SD. One-way ANOVA with the Tukey’s multiple comparison test (A) were used for statistical analysis. **P* < 0.05, ***P* < 0.01.

**Figure S3.**

A	KEGG analysis of the down-regulated mRNAs in lnc-HZ05-overexpressed Swan 71 cells vs control cells.

B, C The protein levels of TGFβ2, TGFβR2, Smad3, and pSmad3 in HTR-8/SVneo cells with lnc-HZ05 overexpression or knockdown and their relative quantification.

D	The protein levels of TGFβ2, Smad3, and pSmad3 in HTR-8/SVneo cells with co-overexpression of lnc-HZ05 and TGFβ2 and their relative quantification.

E	Transwell assay analysis of the migration/invasion of HTR-8/SVneo cells with lnc-HZ05 overexpression or knockdown and their relative quantification. Scale bars, 100 μm.

F	Transwell assay analysis of the migration/invasion of HTR-8/SVneo cells with co-overexpression of lnc-HZ05 and TGFβ2 and their relative quantification. Scale bars, 100 μm.

G	The protein levels of TSPAN4 in Swan 71 or HTR-8/SVneo cells with lnc-HZ05 overexpression and its relative quantification.

All results are representative data from three independent experiments. Data are presented as mean ± SD. The unpaired two-tailed Student’s t-test (C, E and G) and one-way ANOVA with the Tukey’s multiple comparison test (C-F) were used for statistical analysis. **P* < 0.05, ** *P* < 0.01, *** *P* < 0.001, **** *P* < 0.0001.

**Figure S4.**

A	The mRNA levels of FOXP3 in Swan 71 cells with FOPX3 overexpression or knockdown.

B	The mRNA levels of TGFβ2 in HTR-8/SVneo cells with FOPX3 overexpression or knockdown.

C	IP assay analysis of the protein interactions between TGFβ2 and TGFβR2 in Swan 71 cells.

D-G IP assays using using identical but limited TGFβ2 or TGFβR2 antibody showed the protein levels of the immunoprecipitated TGFβR2 or TGFβ2, respectively, in HTR-8/SVneo cells with lnc-HZ05 overexpression or knockdown and their relative quantification, with the protein levels of TGFβ2 and TGFβR2 in cell lysates.

H-J Immunofluorescence of TGFβ2 (green) and TGFβR2 (red) in Swan 71 cells with lnc-HZ05 overexpression or knockdown and their relative quantification. Scale bar, 10 μm.

K The mRNA levels of lnc-HZ05 in Swan 71 or HTR-8/SVneo cells with lnc-HZ05 overexpression and Rnase A treatment.

L	IP assays using using identical but limited TGFβR2 antibody showed the protein levels of the immunoprecipitated TGFβ2 in HTR-8/SVneo cells with lnc-HZ05 overexpression and Rnase A treatment and its relative quantification, with the protein levels of TGFβ2 and TGFβR2 in cell lysates.

M-N Immunofluorescence of TGFβ2 (green) or TGFβR2 (green) and lnc-HZ05 (red) in Swan 71 cells. Scale bar, 10 μm.

O	The levels of lnc-HZ05 enriched by TGFβ2 or TGFβR2 in the first and second rounds of RIP-re-RIP assays in HTR-8/SVneo cells.

P-Q	IP assays using using identical but limited Flag antibody showed the protein levels of TGFβR2 or TGFβ2 immunoprecipitated by Flag-labeled wild-type (wt) or mutant (mut) TGFβ2 or TGFβR2, respectively, in HTR-8/SVneo cells and their relative quantification, with the protein levels of Flag, TGFβ2, and TGFβR2 in cell lysates.

All results are representative data from three independent experiments. Data are presented as mean ± SD. The unpaired two-tailed Student’s t-test (A, B, F, G and O) and one-way ANOVA with the Tukey’s multiple comparison test (A, B, F, G, J, K, L and O) were used for statistical analysis. **P* < 0.05, ***P* < 0.01, ****P* < 0.001, *****P* < 0.0001.

**Figure S5.**

A	IP assays using identical but limited TGFβR2 antibody showed the protein levels of the immunoprecipitated TGFβ2 in HTR-8/SVneo cells with overexpression of lnc-HZ05, HZ05-S1, lnc-HZ05-S2, or lnc-HZ05-S3 and its relative quantification.

B, C	The protein levels of TGFβ2, Smad3, pSmad3, and NDST1 in HTR-8/SVneo cells with overexpression of lnc-HZ05, HZ05-S1, lnc-HZ05-S2, or lnc-HZ05-S3 and their relative quantification.

D	Transwell assay analysis of the migration/invasion of HTR-8/SVneo cells with overexpression of lnc-HZ05, HZ05-S1, lnc-HZ05-S2, or lnc-HZ05-S3 and their relative quantification. Scale bars, 100 μm.

E	The mRNA levels of DNMT1 in HTR-8/SVneo cells with DNMT1 knockdown or overexpression.

F	IP assays using identical but limited TGFβR2 antibody showed the protein levels of the immunoprecipitated TGFβ2 in HC and RM villous tissues and its relative quantification, with the protein levels of TGFβ2 and TGFβR2 in tissue lysates (each n = 6).

All results are representative data from three independent experiments. Data are presented as mean ± SD. The unpaired two-tailed Student’s t-test (D-F) and one-way ANOVA with the Tukey’s multiple comparison test (A, C and D) were used for the correlation analysis. **P* < 0.05, ** *P* < 0.01, *** *P* < 0.001, **** *P* < 0.0001.
